# Pitfalls in quantifying exploration in reward-based motor learning and how to avoid them

**DOI:** 10.1007/s00422-021-00884-8

**Published:** 2021-08-02

**Authors:** Nina M. van Mastrigt, Katinka van der Kooij, Jeroen B. J. Smeets

**Affiliations:** Department of Human Movement Sciences, Vrije Universiteit Amsterdam, Amsterdam, The Netherlands

**Keywords:** Motor learning, Variability, Exploration, Reinforcement, Reward, Trial-by-trial analysis

## Abstract

When learning a movement based on binary success information, one is more variable following failure than following success. Theoretically, the additional variability post-failure might reflect exploration of possibilities to obtain success. When average behavior is changing (as in learning), variability can be estimated from differences between subsequent movements. Can one estimate exploration reliably from such trial-to-trial changes when studying reward-based motor learning? To answer this question, we tried to reconstruct the exploration underlying learning as described by four existing reward-based motor learning models. We simulated learning for various learner and task characteristics. If we simply determined the additional change post-failure, estimates of exploration were sensitive to learner and task characteristics. We identified two pitfalls in quantifying exploration based on trial-to-trial changes. Firstly, performance-dependent feedback can cause correlated samples of motor noise and exploration on successful trials, which biases exploration estimates. Secondly, the trial relative to which trial-to-trial change is calculated may also contain exploration, which causes underestimation. As a solution, we developed the additional trial-to-trial change (ATTC) method. By moving the reference trial one trial back and subtracting trial-to-trial changes following specific sequences of trial outcomes, exploration can be estimated reliably for the three models that explore based on the outcome of only the previous trial. Since ATTC estimates are based on a selection of trial sequences, this method requires many trials. In conclusion, if exploration is a binary function of previous trial outcome, the ATTC method allows for a model-free quantification of exploration.

**Supplementary Information:**

The online version contains supplementary material available at 10.1007/s00422-021-00884-8.

## Introduction

People are able to learn a movement based on binary success feedback only (Cashaback et al. 2019; Codol et al. 2018; Izawa and Shadmehr 2011; Pekny et al. 2015; Therrien et al. 2016, 2018; Uehara et al. 2019; van der Kooij and Smeets 2018). This so-called reward-based motor learning requires exploration (Dhawale et al. 2019; Sutton and Barto 2017): if the only feedback you receive on a movement is on success or failure, you have to explore to find out which movement leads to success. Hence, understanding the mechanism of reward-based motor learning requires understanding of how much a learner explores. Understanding how much a learner explores could be done by fitting a reward-based motor learning model to behavioral data, but model fitting is a difficult process in which parameter estimates may be influenced by each other (Cheng and Sabes 2006). Alternatively, as exploration leads to variability, one could use movement variability as a way to quantify exploration. Exploration is, however, not the only source of movement variability: another source of movement variability is motor noise, which we consider to include all inevitable noise. How can those two sources of variability be separated?

We developed a method for quantifying exploration in the presence of motor noise (van Mastrigt et al. 2020). Like Therrien et al. (2016, 2018), Cashaback et al. (2019) and Dhawale et al. (2019), we assumed that motor noise and exploration are two independent random processes of which the variances can thus be summed to total observed variance. Based on this assumption, we separated the variability caused by motor noise and the variability caused by exploration. We estimated the motor noise from the variability following successful trials, assuming that there is no reason to explore following successful trials. Second, we estimated the total variability following non-successful trials, assuming that in this case, there is a reason to explore. Third, we estimated the contribution of exploration variability to this total variability by subtracting the estimate of motor noise from the total variability following non-successful trials. A common measure of variability is the variance. As we are interested in learning, and the variance is sensitive to a shift in the mean of a signal, we quantified variability by a detrended alternative: the median squared trial-to-trial change (for details, see 12). In this trial-to-trial change (TTC) method, exploration is estimated as the median squared trial-to-trial change following non-successful trials minus the median squared trial-to-trial change following successful trials.

Any new method requires validation, so the question is: Does the TTC method indeed measure exploration? Validating this method is especially important since we developed the TTC method in a task with random reward feedback, whereas the method is targeted at studying exploration during learning. For unraveling the relation between exploration and learning, it is important that the quantification of exploration is not confounded by learning. A learner aims for an improvement in performance by learning on a trial-to-trial basis: following an instance of reward feedback, the intended behavior can be corrected or updated. Learning on a longer time scale can then be observed as systematic changes in the mean behavior. As exploration in the TTC method is estimated based on trial-to-trial changes rather than the variance of a signal, we expect it to be insensitive to a shift in the mean of a signal. Here, we aim to validate the TTC method by testing the relation between known and estimated exploration, as well as how this relation depends on the amount of learning. To do so, we will simulate reward-based motor learning, so that we know the motor noise and exploration variances. Because the exploration is known in the simulations, we can investigate how well the TTC estimate of exploration captures the exploration that was put in the model.

To test how sensitive the TTC exploration measure is to learning, we need to specify how exploration is used in learning. We use four models to simulate reward-based motor learning: the models of Therrien et al. (2016, 2018), Cashaback et al. (2019) and Dhawale et al. (2019). These models have all been fitted to behavioral data, are similar in structure but have interesting differences in how they learn. All models describe how a movement is constructed based on an intended movement and the addition of the two sources of movement variability: exploration and motor noise. The target amplitude and reward criterion determine when a movement is considered successful and will be rewarded. Depending on this reward feedback, the models may learn by adjusting the intended movement on the next trial. Most models assume that the size and direction of the adjustments are based on a fraction of previous exploration (Cashaback et al. 2019; Dhawale et al. 2019; Therrien et al. 2016, 2018). Input to the models thus consists of task parameters—a target amplitude and reward criterion—and of learner parameters describing the learning fraction and the variances of motor noise and exploration. All four models have successfully been fitted to experimental data, either of humans performing a reinforcement visuomotor rotation task (Cashaback et al. 2019; Therrien et al. 2016, 2018) or of rats performing a reinforcement joystick angle press task (Dhawale et al. 2019). Although presented in different terminology, the models have a rather similar structure. This allows us to translate the models into a common terminology to facilitate comparison between models. As we have multiple models describing learning, a second aim is to test how sensitive our measure of exploration is to learner and task parameters in the model. As we will see in the results, the TTC method has severe limitations. To mitigate these limitations, we will therefore present a revised method: the additional trial-to-trial change (ATTC) method.

In summary, we proposed the TTC method for solving the computational problem of estimating exploration as the total variability following non-successful trials minus variability following successful trials. Here, we aim to validate this method by testing how sensitive its exploration measure is to learning according to mechanisms proposed by various models. We will simulate learning from binary reward feedback in a one-dimensional task. Model inputs are a target amplitude, a reward criterion, a learning parameter and parameters describing the variances of motor noise and exploration. We will vary one model parameter at a time, while keeping the other parameters fixed. This way, we explore how well the TTC method captures the actual exploration in a variety of learning conditions. Based on the results, we reformulated the TTC method into the ATTC method that better captured the modeled exploration.

## Methods

**Fig. 1:**
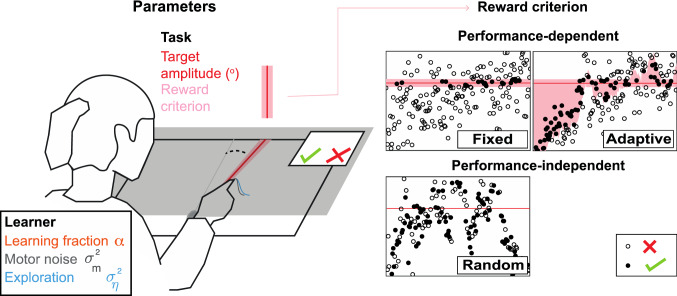
Example task and simulation parameters. The example illustrates a class of motor learning tasks involving binary reward feedback based on one dimension of the movement, with no feedback on error size and sign. Left panel: simulation parameters consist of two task parameters and three learner parameters. Participants aim for a target but might end up somewhere else due to motor noise and exploration in their movement. Upon the next attempt, the learner may adjust her aim point with a learning fraction. The gray screen blocks the sight of the hand, so that task feedback is limited to binary reward feedback. Right panel: three types of the reward criterion task parameter. A fixed reward criterion only rewards movements at the target. An adaptive reward criterion additionally rewards movements that are closer to the target than the mean or median of the past five attempts. A random reward criterion randomly rewards 50% of the movements randomly, independent of performance

Using four reinforcement learning models, we will simulate learning a one-dimensional task based on binary reward feedback only, resembling common experimental tasks such as used by (Cashaback et al. 2019; Therrien et al. 2016, 2018). We restrict our simulations to a task with a one-dimensional outcome, for example, reaching a target at a certain angle (Fig. 1). The learner behaves according to a certain learning model and is defined by the motor noise, exploration and learning fraction. The task is defined by the target amplitude and the criterion based on which the learner is rewarded. This way, we use five parameters in our simulations. Based on our recommendation (van Mastrigt et al. 2020), the task consists of 500 trials.

### Learning models

**Fig. 2:**
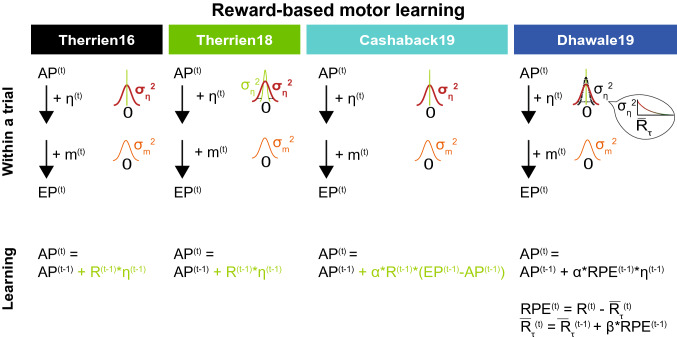
Learning models translated into terminology similar to Van Beers (van Beers 2009). See Table 1 for terminology. The top part describes the construction of a movement within a trial. The bottom part describes learning from trial to trial. Bold red lines indicate the situation following a non-successful trial; thinner green lines indicate the situation following a successful trial. In all models, an endpoint is constructed by adding exploration and motor noise to the aim point. The actual observable behavior is the endpoint, which is then rewarded or non-rewarded. Learning always involves adjusting the aim point. This might consist of updating the aim point only following success and not following failure (Therrien16, Therrien18, Cashaback19), but it might be that the aim point is also corrected following failure (Dhawale19)

We used four models to simulate reward-based motor learning: the models of Therrien et al. (2016) (Therrien16), Therrien et al. (2018) (Therrien18), Cashaback et al. (2019) (Cashaback19) and Dhawale et al. (2019) (Dhawale19) (Fig. 2). They all incorporate the same two sources of movement variability: motor noise and exploration. The variance of total, observable variability is considered the sum of the variances of motor noise ($\sigma_{m}^{2}$) and exploration ($\sigma _{\eta }^{2}$).

All models

1 $$ \sigma _{{total}}^{2}  = ~\sigma _{m}^{2}  + \sigma _{\eta }^{2} $$

To facilitate comparison between the reinforcement learning models, we phrased all models in terms of aim points and endpoints, similar to the terminology employed in Van Beers (van Beers 2009) (Fig. 2) (Table 1). The aim point refers to an intended movement, whereas the endpoint refers to the actual, executed movement. We furthermore refer to all inevitable variability that cannot be assessed internally as motor noise. Note that the planned aim point correction model of Van Beers (van Beers 2009) (vanBeers09 model) differs in two aspects from the reinforcement learning models. Firstly, the vanBeers09 model describes learning from error feedback, instead of reward feedback. Secondly, the vanBeers09 model describes the construction of an endpoint as the serial addition of two sources of motor noise, namely planning noise and execution noise. The reinforcement models describe the construction of an endpoint as the addition of exploration and motor noise, in unspecified order. How the concepts of planning noise, execution noise, motor noise and exploration relate to each other is an open question which we did not try to answer here. The reason that Van Beers (van Beers 2009) assumes a serial order is that he assumes that the planned aim point rather than the aim point is represented in the brain. In the reinforcement learning models, no such distinction is explicitly made. We therefore translated the “intended reach aim” (Cashaback et al. 2019), “mean movement policy” (Dhawale et al. 2019) and “internal estimate of correct reach angle” (Therrien et al. 2018) into “aim point.”

**Table 1:** Terminology

			Model
Time			
$t$	Trial		All
**Sources of variability**			
$m$, $\sigma _{m}^{2}$	Motor noise	Inevitable variability that is always present. Also called unregulated variability (Dhawale et al. 2019)	Cashaback19
			Dhawale19
			Therrien16
			Therrien18
$\eta$, $\sigma _{\eta }^{2}$	Exploration	Variability that can be added and can be learnt from. Also called regulated variability (Dhawale et al. 2019)	Cashaback19
			Dhawale19
			Therrien16
			Therrien18
$\sigma _{{\eta *}}^{2}$		Input exploration	
$\sigma _{{\eta - }}^{2}$		Exploration estimated following non-successful trials	
$\sigma _{{\eta + }}^{2}$		Exploration estimated following successful trials	
$\widehat{{\sigma _{\eta }^{2} }}$		(A)TTC estimate of exploration	
**Movement generation**			
$AP$	Aim point	Mean of the probability density of movement endpoints given a certain ideal motor command (van Beers 2009)	All
$EP$	End point	Observable movement outcome	All
**Reward-based motor learning**			
$R$	Reward presence or absence	R = 0: no reward	All
		R = 1: reward	
$RPE$	Reward prediction error	Difference between actual reward obtained and predicted reward	Dhawale19
		RPE > 0: Reward obtained	
		RPE < 0: No reward obtained	
$\overline{{R_{\tau } }}$	Low-pass filtered reward history	Low-pass filtered reward history of the τ previous trials	Dhawale19
$\alpha$	Reward-based learning parameter	Learning gain, adjustment fraction	Cashaback19
			Dhawale19
			Therrien16
			Therrien18
$\beta$	Reward rate update fraction	Gain of updating the reward rate estimate ($\overline{{R_{\tau } }}$) with the most recent trial outcome	Dhawale19
${{\tau }}$[tau]	Number of trials in reward history memory window	Inferred memory window for reinforcement on past trials, or the time-scale of the experimentally observed decay of the effect of single-trial outcomes on variability (Dhawale et al. 2019)	Dhawale19

#### Constructing a movement endpoint within a trial

On each trial *t*, a movement endpoint (EP) is constructed based on an aim point (AP), motor noise (m) and exploration (η, the Greek letter èta) (Therrien16, Therrien18, Cashaback19, Dhawale19) (Fig. 2). In the Therrien18 and Dhawale19 models, each trial contains exploration (Eq. 2), whereas in the Therrien16 and Cashaback19 models, the addition of exploration to the aim point depends on reward (R) absence in the previous trial (Eq. 2).

Therrien18, Dhawale19:

2 $$ EP^{{\left( t \right)}}  = AP^{{\left( t \right)}}  + \eta ^{{\left( t \right)}}  + m^{{\left( t \right)}} $$

Therrien16, Cashaback19

3 $$ EP^{{\left( t \right)}}  = AP^{{\left( t \right)}}  + \left( {1 - R^{{\left( {t - 1} \right)}} } \right)~\eta ^{{\left( t \right)}}  + m^{{\left( t \right)}} $$

All models assume that motor noise and exploration are randomly drawn from Gaussian distributions with zero mean and variances of $\sigma _{m}^{2}$ and $\sigma _{\eta }^{2}$. In the Therrien18 and Dhawale19 models, $\sigma _{\eta }^{2}$ changes based on reward history.

#### Learning from trial to trial

On each trial, the aim point is defined based on the exploration and reward in the previous trial (Fig. 2).

Therrien16, Therrien18:

4 $$ AP^{{\left( t \right)}}  = AP^{{\left( {t - 1} \right)}}  + R^{{\left( {t - 1} \right)}} ~\eta ^{{\left( {t - 1} \right)}} $$

Cashaback19:

5 $$ AP^{{\left( t \right)}}  = AP^{{\left( {t - 1} \right)}}  + \alpha ~R^{{\left( {t - 1} \right)}} ~\left( {EP^{{\left( {t - 1} \right)}}  - AP^{{\left( {t - 1} \right)}} } \right) $$

Dhawale19:

6 $$ AP^{{\left( t \right)}}  = AP^{{\left( {t - 1} \right)}}  + \alpha ~RPE^{{\left( {t - 1} \right)}} ~\eta ^{{\left( {t - 1} \right)}} $$

7 $$ RPE^{{\left( t \right)}}  = R^{{\left( t \right)}}  - \bar{R}^{{\left( t \right)}} $$

8 $$ \bar{R}^{{\left( t \right)}}  = \bar{R}^{{\left( {t - 1} \right)}}  + \beta ~RPE^{{\left( {t - 1} \right)}} $$

How learning occurs depends on the information the brain has access to. All models assume that the brain does not “know” the size and direction of motor noise on a trial (m^(t)^). Hence, motor noise cannot be used for learning. In the Therrien18 and Dhawale19 models, the brain “knows” the size and direction of exploration (η^(t)^) and can thus use exploration for learning. More specifically, the aim point is changed with exploration following success (R^(t−1)^ = 1; Therrien16, Therrien18; Eq. 4) or as a function of the reward prediction error (RPE^(t−1)^; Dhawale19; Eq. 6). In the Dhawale19 model, the reward prediction error (RPE^(t)^; Eq. 7) is positive if a trial has been successful (R^(t)^ = 1) and negative if it has not been successful (R^(t)^ = 0). Put differently, the three models that learn from known exploration all *update* ( +) the aim point following successful trials, whereas only the Dhawale19 model *corrects* (-) the aim point following non-successful trials.

The Cashaback19 model assumes that the brain has some information on both motor noise and exploration variability. The model has an estimate of the aim point and partial knowledge of the actual reach which is reflected in the term α*(EP^(t)^ – AP^(t)^) (Eq. 5). Following reward (R^(t−1)^ = 1), the aim point is updated with an estimate of previous motor noise (if R^(t−2)^ = 1), since following reward no exploration is added (Eq. 3), or previous motor noise and exploration (if R^(t−2)^ = 0).

The Dhawale19 and Cashaback19 models contain a learning parameter (α): the aim point is adjusted for the next trial with a fraction α of the exploration in the present trial (Dhawale19) or an estimate of motor noise plus exploration (Cashaback19). The Therrien18 model does not contain an explicit learning parameter: following reward, the aim point is updated with the full exploration (i.e., α = 1).

Two of the four models (Therrien18, Dhawale19) propose that exploration depends on the history of obtained rewards. In these models, reward history determines the variance of the distribution from which exploration is drawn ($\sigma _{\eta }^{2} \left( t \right)$). In both models, a history associated with less reward results in a higher exploratory variance than a history with more reward. In the Therrien18 model, only the presence of reward on the previous trial (R^(t−1)^) determines whether exploration on trial t is drawn from a normal distribution with a smaller variance following successful trials ($\sigma _{{\eta  + }}^{2}$) or larger variance following non-successful trials ($\sigma _{{\eta  - }}^{2}$). In the Dhawale19 model, a low-pass filtered reward history of the τ previous trials ($\overline{{R_{\tau } }} ^{{\left( t \right)}}$) determines $\sigma _{\eta }^{2} \left( t \right)$. In rats, Dhawale19 estimated the time-scale τ to be 5 past trials. This time-scale influences the calculation of the low-pass filtered reward history ($\overline{{R_{\tau } }} ^{{\left( t \right)}}$) via a reward rate update fraction (β) (Online Resource 1). The more trials the reward history is based on (i.e., the larger τ), the less the reward history estimate is influenced by the most recent trial outcome (i.e., the smaller β).

Dhawale19:

9 $$ \beta {{~}} = {{~}}1 - e^{{\frac{{ - 1}}{\tau }}} {{~}} $$

#### Model parameters

In order to test robustness of the TTC method, we vary the value of five input parameters of the four models (Table 2): learner parameters motor noise ($\sigma _{m}^{2}$), exploration ($\sigma _{\eta }^{2}$) and learning parameter (α), and task parameters target movement amplitude and reward criterion. The values of the parameters have been chosen based on the experimental tasks, data and parameters reported by Therrien et al. (2016, 2018), Cashaback et al. (2019) and Dhawale et al. (2019) (Online Resource 2). Each parameter will be varied while keeping the other parameters constant: $\sigma _{m}^{2}$, $~\sigma _{\eta }^{2}$, α and target are set to the median value, and the reward criterion is set to be adaptive based on the mean of the past five trials (Table 2).

**Table 2:** Model parameters used for simulating learning. See Table 1 for abbreviations

Varying values (**default value**)				
Learner parameters			Task parameters	
$\sigma _{m}^{2}$	$\sigma _{{\eta *}}^{2}$†	$\alpha$‡	Target amplitude (units of $\sigma _{m}$)	Reward criterion (*R*(*t*) = 1 if: …)
1	1	0	0	Random:
				50% of trials
4	4	0.1	2	Adaptive (median):
				If EP < target: $median~(EP_{{t - 1:t - 10}} )$ ≤ *EP* ≤ target + 1
				If EP within fixed reward zone: target – 1 ≤ EP ≤ target + 1
				If EP > target: target – 1 ≤ EP ≤ $median~(EP_{{t - 1:t - 10}} )$
**9**	**16**	**0.15**	**4**	**Adaptive (mean):**
				If EP < target: $\overline{{EP}} _{{t - 1:t - 10}}$ ≤ EP ≤ target + 1
				If EP within fixed reward zone: target – 1 ≤ EP ≤ target + 1
				If EP > target: target – 1 ≤ EP ≤ $\overline{{EP}} _{{t - 1:t - 10}}$
16	36	0.2	6	Fixed:
				If EP within fixed reward zone: target – 1 ≤ EP ≤ target + 1
25	64	1	8	Fixed with lower target fraction (target fraction = 2):
				If EP within fixed reward zone: target – 1 ≤ EP ≤ target + 1

† The input $\sigma _{{\eta *}}^{2}$ is equal to the exploratory variance used following non-successful trials in the model of Cashaback19 and Therrien16. Using a variability control function (see Eq. 10, Fig. 3), it defines the two exploratory variances in the Therrien18 model, and a whole range of variances in the Dhawale19 model

‡ The values 0.1, 0.15 and 0.2 are not used in the Therrien16 and Therrien18 models, as their learning parameter is fixed at 1 (Eq. 4)

Instead of a fixed exploratory variability, the Therrien18 and Dhawale19 model have a function that controls this variability based on reward history. In the Therrien18 model, the variability control function is a binary function prescribing a different $\sigma _{\eta }^{2}$ following a successful trial ($\sigma _{{\eta  + }}^{2}$) and following a non-successful trial ($\sigma _{{\eta  - }}^{2}$). In the Dhawale19 model, the variability control function is a continuous function ($\sigma _{\eta }^{2}$($\overline{{R_{\tau } }}$)). We set the parameter τ that defines the time-scale based on which the low-pass filtered reward history is calculated, to the 5 trials that Dhawale et al. (2019) report in rats, which results in a parameter β of 0.18 in Eq. 8.

**Fig. 3:**
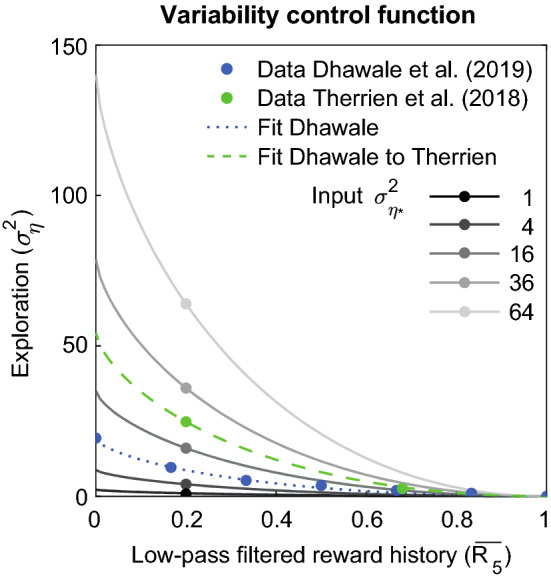
Exploratory variability control functions. The variability control function $\sigma _{\eta }^{2}  = ~c\cdot\left( {\overline{{R_{5} }} ^{{0.7}}  - 1} \right)^{2}$ has been fitted to the variances reported by Dhawale et al. (2019) (c = 19) and scaled to the variances reported by Therrien et al. (2018) (c = 54). This variability control function is used for scaling with the different input values of the exploration parameter $\sigma _{\eta }^{2}$ (Eq. 10)

To define a shared variability control function for the Dhawale19 and Therrien18 models, we first fitted a function through the data of average rat exploratory variability reported in Table 2 (Fig. 3). To scale this variability control function to the $\sigma _{{\eta  + }}^{2}$ and $\sigma _{{\eta  - }}^{2}$ that were reported by Therrien et al. (2018), we simulated 10,000 experiments with a reward rate similar to the experiments of Therrien et al. (2018). Taking the mean of all simulations, this yielded a low-pass filtered reward history $\overline{{R_{5} }} ^{{\left( t \right)}}$ = 0.20 following a non-successful trial, and a low-pass filtered reward history $\overline{{R_{5} }} ^{{\left( t \right)}}$ = 0.68 following a successful trial (Fig. 2). Next, we scaled the variability control function in Fig. 3 to a function including the average value for $\sigma _{{\eta  - }}^{2}$ (at $\overline{{R_{5} }}$ = 0.20) from the two experiments of Therrien et al. (2016, 2018) (Online Resource 2). This resulted in a variability control function (Fig. 3, green line) that can be scaled with input exploration:

Therrien18, Dhawale19

10 $$ \sigma _{\eta }^{2}  = ~2.18\cdot\sigma _{{\eta *}}^{2} \cdot\left( {\overline{{R_{5} }} ^{{0.7}}  - 1} \right)^{2} $$

In our simulations, the input parameter $\sigma _{{\eta *}}^{2}$ of the models is chosen based on the exploratory variances reported following non-successful trials (Online Resource 2). In the Dhawale19 model, $\overline{{R_{5} }} ^{{\left( t \right)}}$ on each trial defines $\sigma _{\eta }^{2}$ on that trial. In the Therrien18 model, $\sigma _{{\eta  - }}^{2}  = \sigma _{{\eta *}}^{2}$ and $\sigma _{{\eta  + }}^{2}  = 0.12\sigma _{{\eta *}}^{2}$. In the two other models $\sigma _{{\eta  - }}^{2}  = \sigma _{{\eta *}}^{2}$, and $\sigma _{{\eta  + }}^{2}  = 0$.

For each combination of input parameters, we run a simulation set of 1000 simulations for all four models. To ensure that all four models run with the same random draws for exploration and motor noise on each simulation within a simulation set, we created two vectors with 500 random draws from $N\left( {0,~1} \right)$: one for the exploration and one for motor noise for each of the 1000 simulations. On each trial $t~$ within that simulation, the *t*-th element of these vectors is multiplied with $\sigma _{\eta }$ or $\sigma _{m}$. All simulations are initialized with an aim point, endpoint, reward, low-pass filtered reward history, reward prediction error, motor noise and exploration of zero.

### Outcome measures

We first explore the relation between input and estimated exploration for all simulation sets. To this end, we calculate the similarity between estimated exploration with input exploration for each simulation expressed as the ratio of estimated exploration variance and input exploration variance. Perfect estimation yields a *similarity ratio (SR)* of 1. To assess the sensitivity of our exploration measure to learning, we next calculate learning as the area under the learning curve divided by total area under the target and visually explore the relation between input-estimated similarity and learning. To assess the sensitivity of our exploration measure to the learning mechanism proposed by the different models, we visually explore whether and how the relation between estimated-input similarity and learning differs between models.

We quantify exploration as total variability following non-successful trials minus the total variability following successful trials (van Mastrigt et al. 2020). In this method, we estimate the variances in Eq. 1 by the median of squared trial-to-trial changes (Eq. 11, numerator). In one dimension, the relation between the mean amplitude of trial-to-trial changes and standard deviation is (Thirey and Hickman 2015): $\overline{{\left| \Delta  \right|}}  = \frac{2}{{\sqrt \pi  }}\sigma$.

As our measure of the validity of the TTC method, we use the ratio between estimated exploration ($\widehat{{\sigma _{\eta }^{2} }}$) and the exploration that was actually present in the simulation ($\sigma _{{\eta *}}^{2}$). We will call this the *similarity ratio (SR):*

11 $$ SR = \frac{{\widehat{{\sigma _{\eta }^{2} }}}}{{\sigma _{{\eta *}}^{2} }} = \frac{{\frac{{{\pi }}}{4}\times a\times \left( {{{\tilde{\delta }}}_{ - }^{2}  - {{\tilde{\Delta }}}_{ + }^{2} {{~}}} \right)}}{{\sigma _{{\eta *}}^{2} }} $$

where ${{\tilde{\Delta }}}_{ + }^{2}$ and ${{\tilde{\Delta }}}_{ - }^{2}$ are the median of squared trial-to-trial changes following successful and non-successful trials, respectively. Since we estimate exploration as the additional variability following non-successful trials, the numerator in Eq. 11 is the difference in input exploration following non-successful and following successful trials. In the Therrien16 and Cashaback19 models, this difference will equal $\sigma _{{\eta *}}^{2}$ since $\sigma _{{\eta  + }}^{2}  = 0$. For those models, the correction factor *a* = 1. In the Therrien18 model, this difference will not equal $\sigma _{{\eta *}}^{2}$ since $\sigma _{{\eta  + }}^{2}$ = 0.12 $\sigma _{{\eta *}}^{2}$. We correct for this with correction factor *a* = 1.14. In the Dhawale19 model, the difference between input exploration following non-successful and following successful trials depends on the low-pass filtered reward history on trials following non-successful and successful trials. The correction factor *a* thus varies between simulations and is calculated based on the average values of the low-pass filtered reward history over all non-successful and successful trials, so that $a = ~\frac{1}{{\overline{{\overline{{R_{5} }} ^{{1.4}} }} _{ - }  - ~\overline{{\overline{{R_{5} }} ^{{1.4}} }} _{ + }  - 2\overline{{\overline{{R_{5} }} ^{{0.7}} }} _{ - }  + ~2\overline{{\overline{{R_{5} }} ^{{0.7}} }} _{ + } }}$.

Learning is achieved by systematic trial-to-trial changes and can shift the mean behavior. It can be characterized by several parameters, such as leaning speed or learning asymptote. For the present purpose (identifying a possible relation between learning and the estimation of exploration), we characterize learning by a single parameter: *learning.* This parameter ranges from 0% for no learning and 100% for instant full learning. To calculate *learning*, we determined a smoothed learning curve as the running average of endpoints over a window of 20 trials, calculated the area under this curve, divided this by the total area under the target and multiplied this with 100%.

## Results

We simulated learning from binary reward feedback in a one-dimensional task with four different reward-based motor learning models. The models are given 500 trials to learn on target movement. Each simulation represents one model learner who has to reach a target a certain amplitude (expressed in units of $\sigma _{m}$) away from baseline performance, while receiving feedback based on a certain reward criterion. For each combination of learning model and parameter set, we performed a set of 1000 simulations. A simulation set can be considered as one experimental task (*target amplitude* & *reward criterion*) performed 1000 times by a learner with a certain variability ($\sigma _{m}^{2}$, $\sigma _{{\eta *}}^{2}$) and learning (α) characteristics. From the simulated behavior, we estimated exploration with the TTC method. To estimate the similarity ratio, we divided estimated exploration by the exploration that we knew was in the models in order to obtain the similarity ratio.

### Model behavior

**Fig. 4:**
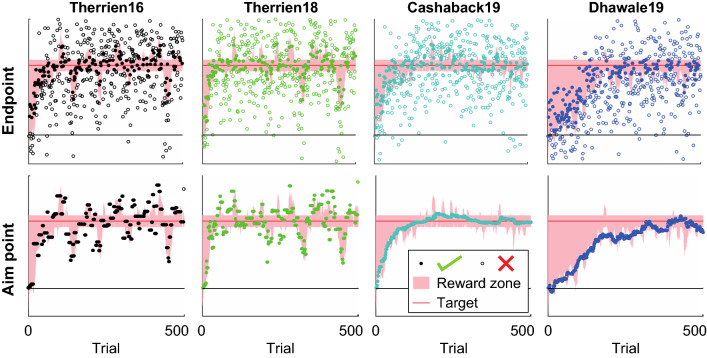
Example learning curves. One example simulation for each model: $\sigma _{m}^{2}  = 9$, $~\sigma _{{\eta *}}^{2}  = 16$, α = 0.15 (for the Cashaback19 and Dhawale19 models), reward criterion = adaptive (mean), target amplitude = 4 $\sigma _{m}$. The top row shows the movement endpoint behavior that the TTC method uses to estimate exploration. Each movement endpoint is composed of an aim point, motor noise and possibly exploration. The bottom row shows the underlying aim points that are adjusted during learning. Non-successful trials are covered largely by successful trials because following failure, the aim point is not (or only slightly: Dhawale19) updated

In general, the model learners reached the target within 500 trials. The example simulations in Fig. 4 were generated with all parameters set to their default value (bold in Table 2). Each learner was initialized at starting location zero and received reward feedback based on endpoint position (Fig. 4, upper row). Endpoints were constructed by adding motor noise and/or exploration to the aim point (middle row) on a trial-by-trial basis. Each model received the same random motor noise draws. Exploration was also based on the same random draws from a normal distribution N(0,1). These draws, however, resulted in different exploration values since they were scaled with $\sigma _{\eta }$, which is defined differently by each model (Fig. 2, 3). Within a simulation the observed endpoint behavior is highly variable: individual learning curves seem peaky and irregular rather than smooth (Fig. 4, upper row). This is in line with experimental observations (e.g., Cashaback et al. 2019; Therrien et al. 2016, 2018). Since in the Therrien16 and Therrien18 models the learning parameter α is set to one by default (Fig. 1, Table 2), these models learn faster with more aim point variability than the other two models that use α = 0.15 by default (Fig. 4, bottom row).

If we average across all 1000 simulations within a simulation set, all combinations of parameters result in smooth learning curves (Online Resource 3). Although the average learning curves seem smooth, learning curves are highly variable across repetitions: the considerable standard deviations in Fig. 10 (Online Resource 3) indicate that in some simulations, the target was reached very soon, and in others later or even never. Learning was not possible with a learning parameter of zero and with the random reward criterion (Online Resource 3).

### The TTC method

**Fig. 5:**
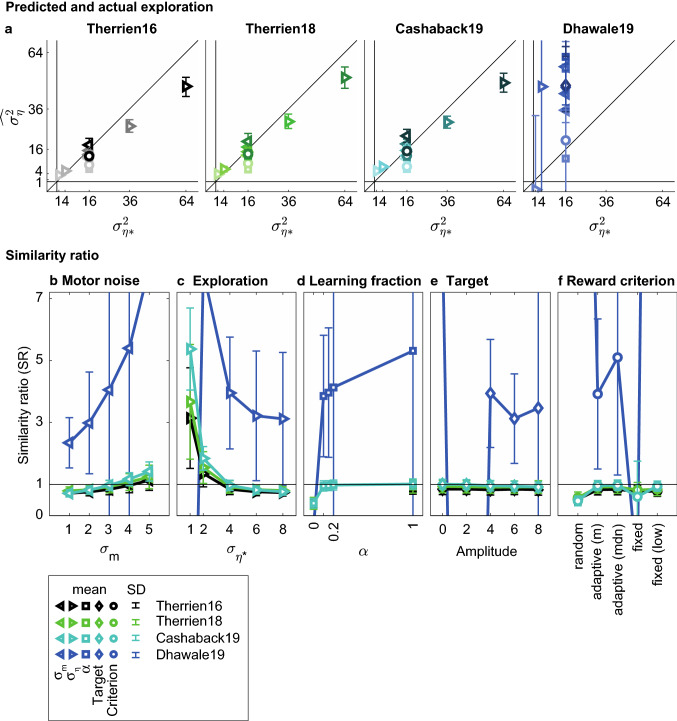
Exploration estimated by the TTC method. Means and standard deviations have been calculated per simulation set of 1000 simulations for the four learning models. Colors indicate the learning model, symbols the parameter that is varied: variance of motor noise ($\sigma _{m}^{2}$) and exploration ($\sigma _{\eta }^{2}$), learning parameter (α), target amplitude and reward criterion. For the Dhawale19 model, various simulations resulted in values outside the plotted range; error bars have only been plotted when the mean was within the axis range. **a** TTC estimates of exploration ($\widehat{{\sigma _{\eta }^{2} }}$) as a function of input exploration ($\sigma _{{\eta *}}^{2}$). The diagonals indicate the unity line, at which estimates are perfect. Note that most simulations have been run with the default exploration $\sigma _{{\eta *}}^{2}  = 16$. **b-f** Similarity ratio ($\widehat{{\sigma _{\eta }^{2} }}/\sigma _{{\eta *}}^{2}$) as a function of the parameters varied. Horizontal lines at SR = 1 indicate perfect estimation

**Fig. 6:**
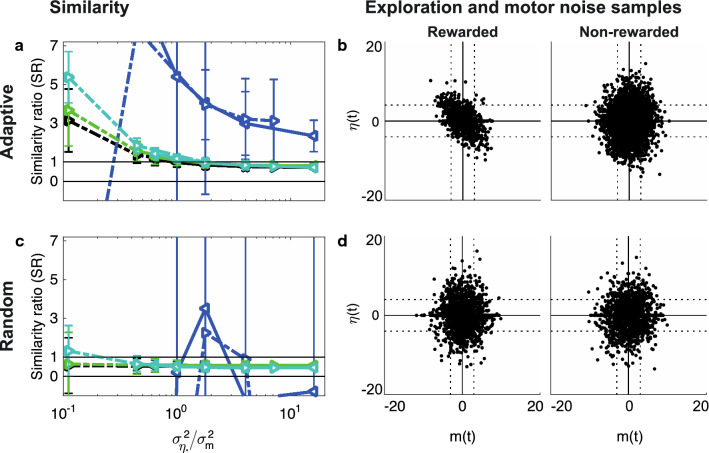
TTC estimation of exploration is sensitive to the balance between exploration and motor noise. We compare model behavior for an adaptive (**a**, **b**) and a random reward criterion (**c**, **d**). **a, c** Similarity ratio as a function of the ratio of exploration and motor noise variance parameters. The horizontal line at SR = 1 indicates perfect estimation of exploration. Symbols and other details as in Fig. 5. **b, d** Draws of motor noise and exploration on successful and non-successful trials in five simulations of a simulation set of the Therrien16 model, resulting in 2500 samples plotted in total for the rewarded and non-rewarded panel together. Except for the reward criterion, other parameters in the simulations have their default values. The data are split based on whether these trials were rewarded or not. Dotted lines indicate $\pm \sigma$

We use the similarity ratio (SR) as a performance measure of the TTC method. It tells us how well the TTC estimate of exploration ($\widehat{{\sigma _{\eta }^{2} }}$) captures the exploration that was present in the simulation ($\sigma _{{\eta *}}^{2}$). A similarity ratio of one indicates that the exploration is perfectly estimated. Figure 5, however, shows that the estimates were far from perfect. Across models, three observations can be made. Firstly, the mean similarity ratios were not close to one for most simulation sets. Secondly, in several cases there was a large variability within a simulation set (large error bars), especially for the Dhawale19 model. Thirdly, similarity ratios depended systematically on the exact parameters of the simulation. Especially important is the result that the TTC estimation of exploration is sensitive to both sources of variability. The TTC method overestimates exploration if it is low or the motor noise high (Fig. 5b,c) and underestimates it if the exploration is high and the motor noise low (Fig. 5b,c). This can be summarized as a sensitivity of the TTC method to the ratio of exploration and motor noise (Fig. 6a). Furthermore, the TTC method is sensitive to the learning parameter α: when α = 0, the TTC method underestimates exploration by 50% as compared to all nonzero values for the learning parameter (Fig. 5d: Therrien16, Therrien18, Cashaback19). Lastly, the TTC method is sensitive to the reward criterion, with a random reward criterion resulting in larger underestimation of exploration as compared to both performance-dependent reward criteria (Fig. 5f). This means that the TTC method is sensitive to both learner and task characteristics. The only parameter that the TTC method is not sensitive to is the target amplitude (Fig. 5e). Between models, the most striking difference is between the Dhawale19 model and the other three models. Firstly, the similarity ratios of the Dhawale19 model deviate more from one than in the other models, both when exploration is overestimated and when it is underestimated. Even negative exploration estimates are found. Secondly, the exploration estimates based on the Dhawale19 learning behavior are highly variable across variations of one parameter. Contrary to the similarity ratios of the other models, patterns in sensitivity to the parameters are less convincing (Fig. 5b,c) or seemingly absent (Fig. 5e,f) for the Dhawale19 model.

The results are puzzling. The estimation of exploration is confounded by learning and the reward criterion used. Some learning seems to be essential in order to estimate exploration well: both when α = 0 and when random reward is provided, exploration is underestimated by about 50%. To understand these results, we used the simplest model: the Therrien16 model. In this model, a learner only explores following failure, and updates with the full exploration following success (Fig. 2). To simplify reasoning, we set both the learning parameter and the level of motor noise to zero, i.e., $\sigma _{m}^{2}  = 0$ and α = 0. This means that the only contributor to trial-to-trial change is exploration. This way, we identified two pitfalls in quantifying exploration with the TTC method.

#### Pitfall 1: Correlated samples of motor noise and exploration

Why are the models so sensitive to the balance between input exploration and motor noise (Fig. 5b,c)? The reason is that performance-dependent reward causes biased samples of motor noise and exploration on successful trials. Although the models generate motor noise and exploration randomly and independently, in the selection of successful trials the random draws are negatively correlated (Fig. 6b), thereby influencing the trial-to-trial changes (Online Resource 4). If exploration drives the movement left of the target, the trial will only be rewarded if motor noise in that trial happens to be in the rightward direction. The correlated samples of motor noise and exploration violate the assumption of independent sources of variability (Eq. 1) that the TTC method is based on. When reward is independent of performance, the correlation disappears. Indeed, also the dependence of the similarity ratio on the ratio of variability sources disappears (Fig. 6c) when the reward criterion is set to random, at least for the Therrien16, Therrien18 and Cashaback19 models.

#### Pitfall 2: Reference trial exploration

**Fig. 7:**
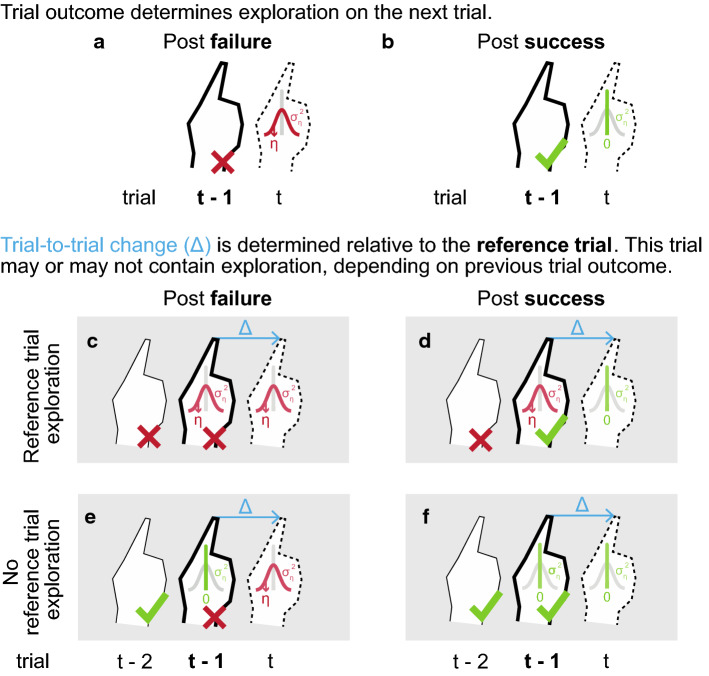
Reference trial exploration causes TTC method to underestimate exploration. Here, we simplify model behavior of the Therrien16 model by assuming that the learner is not noisy ($\sigma _{m}^{2}$ = 0) and does not adjust his aim point following success (α = 0). The only contributor to trial-to-trial change (blue) is hence exploration. The hands indicate different trials over time. The reference trial is indicated with bold lines, the target trial with dotted lines. **a, b** Success or failure determines exploration on the next trial: on trials post-failure (**a**), exploration is drawn from a distribution with variance $\sigma _{{\eta *}}^{2}$ (i.e., $\eta ^{{\left( t \right)}}  \leftarrow ~{{N}}\left( {0,~\sigma _{{\eta *}}^{2} } \right)$). On trials post-success (**b**), exploration is zero ($\eta ^{{\left( t \right)}}  = 0)$. **a-d** Trial-to-trial change is calculated relative to a reference trial, here the successful or non-successful trial. This trial may (**c, d**) or may not (**e, f**) have contained exploration that contributes to the trial-to-trial change. Post-double failure (**a**), trial-to-trial change consists of the difference in exploration between the target and reference trial**.** Post-single failure or success (**b, c**), trial-to-trial change consists of the difference between one exploration draw and zero. Post-double success (**d**), trial-to-trial change is zero

Although random reward results in an estimation of exploration that is insensitive to the size of exploration, estimates are biased: exploration is underestimated with about 50% (Fig. 6c). Apparently, a second problem is present in the TTC method. The TTC method estimates variability based on trial-to-trial changes. The either successful or non-successful trial *t – 1* (Fig. 7a,b) serves as the reference trial relative to which the change in behavior is calculated. However, trial *t – 1* is not a very good reference, as it can contain exploration (if the reference trial is preceded by a successful trial (Fig. 7c,d)) or not (if it is preceded by a non-successful trial (Fig. 7e,f). We estimated this effect with the Therrien16 model, using random reward, a learning parameter α = 0 and the default target amplitude. By setting the level of motor noise to zero, i.e., $\sigma _{m}^{2}  = 0$, the only contributor to trial-to-trial change is exploration (Fig. 7). Whether the learner explores on a trial depends on trial outcome of the previous trial (Fig. 7a,b): post-failure, exploration is drawn from a distribution with $\sigma _{{\eta *}}^{2}$ [i.e., $\eta \left( t \right) \leftarrow ~{{N}}\left( {0,~\sigma _{{\eta *}}^{2} } \right)$], and post-success, exploration is zero ($\eta \left( t \right)$ = 0). The mix between presence and absence of exploration in the reference trial causes the TTC method to underestimate exploration. The TTC method estimates exploration as the variability in all trials post-failure (Fig. 7a,c,e) minus the variability in all trials post-success (Fig. 7b,d,f). For a proper comparison, however, one should ensure that for both types of trial-to-trial changes, the reference trial either contains exploration (c minus d) or no exploration (e minus f).

### The ATTC method

To overcome the two pitfalls related to the TTC method, we developed the ATTC method. As in the TTC method, exploration is estimated by subtracting variability post-non-successful from variability post-successful trials, and variability is calculated based on the median of squared trial-to-trial changes. We made two revisions in the computational method. Firstly, to make the method robust to tasks with performance-dependent feedback, trial-to-trial changes are now calculated relative to the trial before the successful or non-successful trial (trial *t – 2*) instead of the successful or non-successful trial itself (trial *t – 1*). Secondly, to solve the underestimation problem, trial-to-trial change-based variability estimates post-success and post-failure are now subcategorized based on reward history. As exploration on the reference trial is prescribed by reward presence or absence on the previous trial, the subtraction of variability estimates is now performed separately for specific subcategories of reward sequences.

#### Methods

**Fig. 8:**
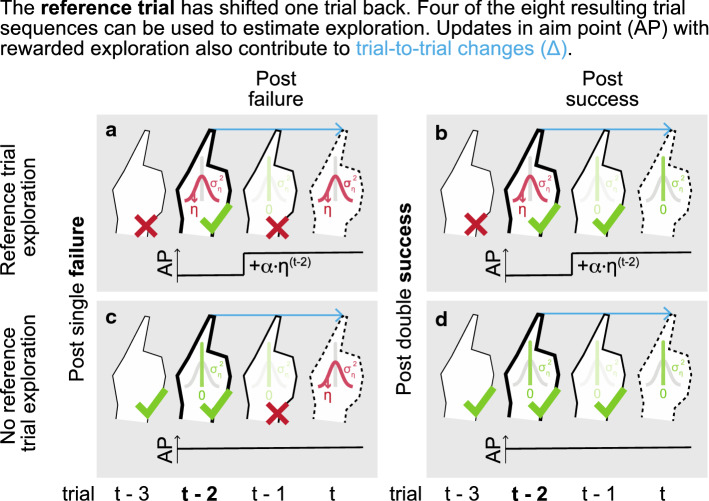
The ATTC method. The ATTC method has been developed based on the Therrien16 model. The ATTC method uses four trial sequences to estimate variability from. The learner only explores post-failure (i.e., $\eta ^{{\left( t \right)}}  \leftarrow ~{{N}}\left( {0,~\sigma _{{\eta *}}^{2} } \right)$; otherwise $\eta ^{{\left( t \right)}}  = 0$). Post-success, the learner learns by updating the aim point (horizontal lines) with a fraction α of the rewarded exploration. In the figure, motor noise is not displayed ($\sigma _{m}^{2}$ = 0). In the ATTC method, the reference trial (in bold) has been changed from the (non-)successful trial to the trial preceding it. Exploration is estimated twice, by subtracting variability estimates calculated from trial-to-trial changes of trial sequences post-single failure and post-double success pairwise, depending on reference trial exploration (a, b vs. c, d). A weighted average of the two is used to obtain one exploration estimate. **a, c** Trial sequences ending with a single failure (*t – 1*). If trial t-3 was non-successful (**a**), trial-to-trial change is a difference between two exploration draws: exploration on the target trial and a fraction (1 – α) of the reference trial exploration. If trial *t – 3* was successful (**c**), trial-to-trial change only consists of exploration on the target trial. **b, d.** Trial sequences ending with double success to calculate post-success variability from. If the trial *t – 3* was non-successful (**b**), trial-to-trial change is a fraction (*1 – α*) of the reference trial exploration. If the first trial was successful (**d**), trial-to-trial change is zero

The ATTC method uses two exploration estimates. Both are obtained by subtracting the variability estimates post-double success from the variability estimates post-single failure (Fig. 8a, Table 3). As we are using trial t-2 as the reference, we will consider sequences of three trials: the (non-)successful trial *t – 1*, and the two trials preceding it. We will use the notation $\widetilde{{{{\Delta }}^{2} }}_{{ + ,p{\mathbf{q}}1}}$ for the median of squared trial-to-trial changes post-success and $\widetilde{{{{\Delta }}^{2} }}_{{ - ,p{\mathbf{q}}0}}$ for the median of squared trial-to-trial changes post-failure. The indices *p* and *q* correspond to trials *t – 2* and *t – 1*. Indices are one if the trial was and zero if the trial was not successful.

**Table 3:** The ATTC method. Each three-trial reward sequence results in a different variance estimate, depending on the contribution of exploration draws ($\eta$) and learning ($\alpha$) to the trials. Only sets C (corresponding to panels a, b in Fig. 8) and D (corresponding to panels c, d in Fig. 8) result in an estimate of exploration variance and are used in Eq. 12

Set	Post-failure sequences			Post-success sequences			Post-failure – post-success	
	Sequence	Estimate	Trial-to-trial change (${{\Delta }}$)	Sequence	Estimate	Trial-to-trial change (${{\Delta }}$)	Exploration estimate ($\widehat{{\sigma _{\eta }^{2} }}$)	Weight
A	$\widetilde{{{{\Delta }}^{2} }}_{{ - ,0{\boldsymbol{0}}0}}$	${{\sigma }}_{{{{\Delta }}\eta }}^{2}$	$~\eta \left( t \right) - \eta \left( {t - 2} \right)$	$\widetilde{{{{\Delta }}^{2} }}_{{ - ,0{\boldsymbol{0}}0}}$	${{\sigma }}_{{{{\Delta }}\eta }}^{2}$	$\alpha *\eta \left( {t - 1} \right) - \eta \left( {t - 2} \right)$	$\widetilde{{{{\Delta }}^{2} }}_{{ - ,0{\boldsymbol{0}}0}} - ~\widetilde{{{{\Delta }}^{2} }}_{{ + ,0{\boldsymbol{0}}1}} \overset{\wedge}{=}~0$	0
B	$\widetilde{{{{\Delta }}^{2} }}_{{ - ,1{\boldsymbol{0}}0}}$	$~{{\sigma }}_{\eta }^{2}$	$\eta \left( t \right)$	$\widetilde{{{{\Delta }}^{2} }}_{{ + ,1{\boldsymbol{0}}1}}$	${{\sigma }}_{\eta }^{2}$	$\alpha *\eta \left( {t - 1} \right)$	$\widetilde{{{{\Delta }}^{2} }}_{{ - ,1{\boldsymbol{1}}0}} - ~\widetilde{{{{\Delta }}^{2} }}_{{ + ,1{\boldsymbol{0}}1}} \overset{\wedge}{=}~0$	0
C	$\widetilde{{{{\Delta }}^{2} }}_{{ - ,0{\boldsymbol{1}}0}}$	$~{{\sigma }}_{{{{\Delta }}\eta }}^{2}$	$\eta \left( t \right) - \left( {1 - \alpha } \right)*\eta \left( {t - 2} \right)$	$\widetilde{{{{\Delta }}^{2} }}_{{ + ,0{\boldsymbol{1}}1}}$	${{\sigma }}_{\eta }^{2}$	$\left( {1 - \alpha } \right)*\eta \left( {t - 2} \right)$	$\widetilde{{{{\Delta }}^{2} }}_{{ - ,0{\boldsymbol{1}}0}} - ~\widetilde{{{{\Delta }}^{2} }}_{{ + ,0{\boldsymbol{1}}1}}$ $\overset{\wedge}{=}~{{\sigma }}_{\eta }^{2}$	3
D	$\widetilde{{{{\Delta }}^{2} }}_{{ - ,1{\boldsymbol{1}}0}}$	${{\sigma }}_{\eta }^{2}$	$\eta \left( t \right)$	$\widetilde{{{{\Delta }}^{2} }}_{{ + ,1{\boldsymbol{1}}1}}$	0	$0$	$\widetilde{{{{\Delta }}^{2} }}_{{ - ,1{\boldsymbol{1}}0}} - ~\widetilde{{{{\Delta }}^{2} }}_{{ + ,1{\boldsymbol{1}}1}} \overset{\wedge}{=}~{{\sigma }}_{\eta }^{2}$	1

Based on the eight possible trial sequences, we can create four variability estimates (Table 3) by pairwise subtraction of variability estimates post-failure and post-success. Only the two differences between variability post-single failure and post-double success are related to exploration (Eq. 12). Estimate C is based on subtracting variability estimates that both contain reference trial exploration ($\widetilde{{{{\Delta }}^{2} }}_{ - ,0{\boldsymbol{1}0}}  - ~\widetilde{{{{\Delta }}^{2} }}_{+ ,0{\boldsymbol{1}1}}$) and estimate D is based on subtracting variability estimates that both do not contain reference trial exploration ($\widetilde{{{{\Delta }}^{2} }}_{ - ,1{\boldsymbol{1}0}}  - ~\widetilde{{{{\Delta }}^{2} }}_{ + ,1{\boldsymbol{1}1}}$) (Fig. 8). Due to the subtraction, both exploration estimates are based on only one exploration draw per trial-to-trial change. The median of squared trial-to-trial changes based on one exploration draw yields an estimate of the variance of exploration itself ($\widetilde{{{{\Delta }}^{2} }} \overset{\wedge}{=}~{{\sigma }}_{\eta }^{2}$) (Table 3). In this case, the relation between the median of squared trial-to-trial changes and the variance is ${{\sigma }}^{2}  = 2.19 \cdot \widetilde{{{{\Delta }}^{2} }}$ (Online Resource 5). The factor 2.19 replaces the Thirey–Hickman factor of $\pi /4$ that was used in the TTC method (Eq. 11) and that was based on two random draws per trial-to-trial change.

As the estimates A and B are unrelated to exploration, only the two exploration estimates C and D are weighted based on 1) the minimum number of trials that each estimate is based on ($w_{{{{Ntrials}}}}$), and 2) the amount of exploration draws each estimate is based on ($w_{{{{N}}\eta }}$, see Table 3) (Eq. 12.

12 $$  \widehat{\sigma _{\eta }^{2} } = a \cdot \frac{w_{{\text{N}}\eta ,C}  \cdot w_{{\text{Ntrials}},{\text{C}}}  \cdot 2.19 \cdot \left( \widetilde{{\Delta ^{2} }}_{ - ,0{\boldsymbol{1}0}}  - ~\widetilde{{\Delta ^{2} }}_{{ + ,0{\boldsymbol{1}1}} } \right) + ~w_{{{\text{N}}\eta ,D}}  \cdot w_{{{\text{Ntrials}},{\text{D}}}} ~ \cdot 2.19 \cdot \left( \widetilde{{\Delta ^{2} }}_{- ,1{\boldsymbol{1}0}}  - ~\widetilde{{\Delta ^{2} }}_{{ + ,1{\boldsymbol{1}1}} } \right)}{{w_{{{\text{N}}\eta ,C}} ~ \cdot w_{{{\text{Ntrials}},{\text{C}}}}  + ~w_{{{\text{N}}\eta ,D}}  \cdot w_{{{\text{Ntrials}},{\text{D}}}} }} $$

Per exploration estimate, the weights are as follows: $w_{{{{Ntrials}},{{C}}}}  = ~{{min}}\left( N0{\boldsymbol{1}0},N0{\boldsymbol{1}1} \right)$, $w_{{{{Ntrials}},{{~D}}}}  = ~{{min}}\left( N1{\boldsymbol{1}0},N1{\boldsymbol{1}1} \right)$, $w_{{{{N}}\eta ,C}}$ = 3 and $w_{{{{N}}\eta ,D}}$ = 1. The correction factor $a$ is the same as in Eq. 11 and corrects for the presence of exploration that is present after successful trials in some of the models (a = 1 (Therrien16, Cashaback19), 1.14 (Therrien18), depends on reward history (Dhawale19)). This way, the *similarity ratio (*$SR$*)* between input ($\sigma _{{\eta *}}^{2}$) and estimated exploration ($\widetilde{{\sigma _{\eta }^{2} }}$) is calculated in a similar way as for the TTC method (Eq. 11). The only exception is that the ATTC method does not need a correction with the Thirey–Hickman factor (Online Resource 5).

#### Results

**Fig. 9:**
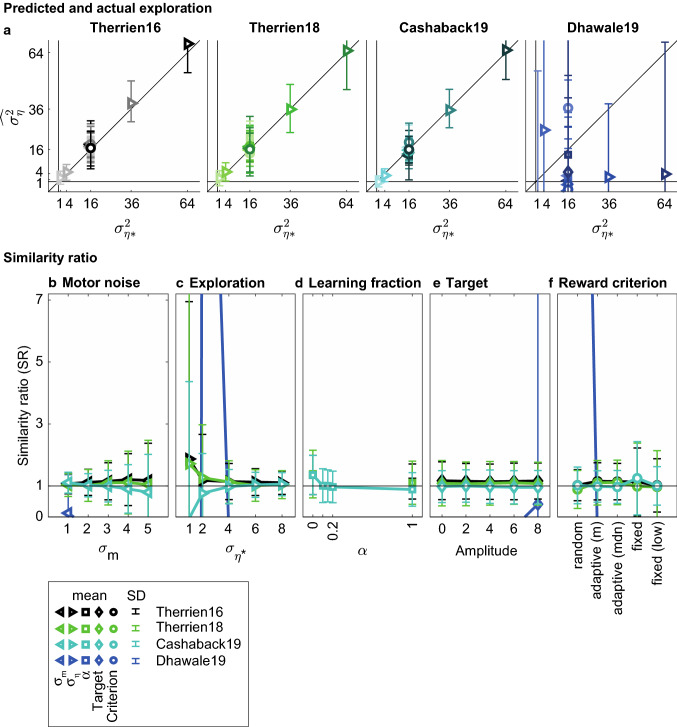
Exploration estimated by the ATTC method. Means and standard deviations have been calculated per simulation set of 1000 simulations for the four learning models. Colors indicate the learning model, symbols the parameter that is varied: variance of motor noise ($\sigma _{m}^{2}$) and exploration ($\sigma _{\eta }^{2}$), learning parameter (α), target amplitude and reward criterion. For the Dhawale19 model, various simulations resulted in values outside the plotted range; error bars have only been plotted when the mean was within the axis range. **a** ATTC estimates of exploration ($\widehat{{\sigma _{\eta }^{2} }}$) as a function of input exploration ($\sigma _{{\eta *}}^{2}$). The diagonals indicate the unity line, at which estimates are perfect. Note that most simulations have been run with the default exploration $\sigma _{{\eta *}}^{2}  = 16$. **b-f** Similarity ratio ($\widehat{{\sigma _{\eta }^{2} }}/\sigma _{{\eta *}}^{2}$) as a function of the parameters varied. Horizontal lines at SR = 1 indicate perfect estimation

To inspect performance of the revised ATTC method, we use the similarity ratio (SR) between the revised ATTC estimate of exploration ($\widehat{{\sigma _{\eta }^{2} }}$) and the exploration that was actually present in the simulation ($\sigma _{{\eta *}}^{2}$). Comparison of Fig. 9 with Fig. 5 informs us that the revised ATTC method has improved estimation of exploration: both the systematic underestimation of exploration and the sensitivity to the balance between motor noise and exploration (Fig. 2) are no longer present. We make three observations from Fig. 9. Firstly, most similarity ratios are close to one for three of the four models, indicating that the revised ATTC method estimates the actual exploration quite well on average. Apparently, a strength of the ATTC method is that it is relatively insensitive to the learning model used. As in the TTC method, similarity ratios of the Dhawale19 model are far off. Secondly, despite the improvement in validity, error bars have increased. This reduction in reliability is not surprising since the ATTC method uses about half of the trial-to-trial changes. The reliability of similarity ratios scales with the square root of the number of simulated trials. Thirdly, in the ATTC method both pitfalls have been solved to a large degree, but still some sensitivity to the balance between variability sources can be observed (Fig. 9f). As this effect seems opposite to the effect found in Fig. 6a, this might, however, reflect something else. In addition to testing the validity of the ATTC method, we visually inspected whether a relation exists between ATTC exploration estimates and learning. We found no clear relation between similarity ratio and learning for the four models (Online Resource 6).

## Discussion

We previously developed a method for quantifying exploration as the additional variability following non-successful trials as compared to successful trials (van Mastrigt et al. 2020). Here, we tested whether this method could be applied to reward-based motor learning. Using four existing models of reward-based motor learning, the method showed to be sensitive to both learner and task characteristics. We identified two pitfalls in quantifying exploration based on trial-to-trial changes. The first is that the use of performance-dependent feedback can cause correlated samples of motor noise and exploration on successful trials. This biases estimates of exploration depending on the balance between motor noise and exploration. The second pitfall is related to the presence of exploration in the trial relative to which trial-to-trial change is calculated. As some reference trials do and others do not contain exploration, this results in an underestimation of exploration. In a newly developed additional trial-to-trial change (ATTC) method, we circumvent these problems by moving the reference trial one trial back and subtracting trial-to-trial changes following specific sequences of trial outcomes. This results in valid exploration estimates for three of the four reward-based motor learning models that we used.

The results indicate that ATTC estimates of exploration are on average similar to exploration put into the model. However, exploration estimates for the Dhawale19 model deviate in a non-consistent manner from input exploration. A major difference between the Dhawale19 model and the other models is its complexity. The model prescribes a range of exploration variances depending on a reward history of past trial outcomes rather than two exploration variances depending on previous trial outcome. Also, how much the aim point is adjusted depends on a reward prediction error rather than binary trial outcome. Moreover, the Dhawale19 model has been developed based on rat data, rather than human data. With regard to the increased complexity, it is not surprising that the Dhawale19 model is the model with the poorest results. Estimating exploration well for learners that learn according to the Dhawale19 model would require a more sophisticated method, in which not one exploration variance is estimated but the full variability control function. Another issue with the ATTC method is the decreased number of trial-to-trial changes that exploration estimates are based on. Instead of using all trial-to-trial changes post-success and post-failure, specific trial sequences are selected. Indeed, our results show higher standard deviations for the ATTC exploration estimates than when simply comparing variability following success and failure as in (van Mastrigt et al. 2020) (Fig. 4 vs Fig. 9). This can be solved by increasing the number of trials in an experiment. Our code is available to run simulations to determine the number of trials needed to obtain a desired reliability.

Reinforcement learning theory predicts exploitation of successful actions and exploration following non-successful actions (Sutton and Barto 2017). Indeed, experimental findings consistently show higher variability following non-successful than following successful trials (Cashaback et al. 2019; Chen et al. 2017; Pekny et al. 2015; Sidarta et al. 2018; Therrien et al. 2016, 2018; Uehara et al. 2019; van der Kooij et al. 2018; van Mastrigt et al. 2020). The addition of more exploration following non-successful trials than following successful trials is a shared feature of the four models and the method presented in (van Mastrigt et al. 2020). This apparently simple shared principle, however, was not enough to obtain valid exploration estimates: the estimates differed much from exploration that was actually present in the models, and also differed much between the models. These results pointed us at two issues with estimating variances from trial-to-trial changes and systematic reward.

The first pitfall that we identified is that performance-dependent reward can introduce correlations between samples of motor noise and exploration. If one wants to evoke learning, one will typically reward trials that have a small or smaller deviation from a target than non-rewarded trials. This induces a constraint on the variability on rewarded trials. Hence, when motor noise on a trial is large, exploration must be small in order to obtain reward. This results in a negative correlation between samples of motor noise and exploration. This is problematic because we assume, in line with the literature (Cashaback et al. 2019; Dhawale et al. 2019; Therrien et al. 2016, 2018), that motor noise and exploration are two independent sources of variability of which the variances can thus be summed to total variability. When this pitfall is not circumvented, the effect of this covariance between motor noise and exploration is that for learners who explore little relative to their motor noise, exploration variance is overestimated. Probably, the cause of this overestimation is an underestimation of motor noise due to the negative covariance term on successful trials. Sensitivity to the balance between exploration and motor noise should be circumvented because this balance has been found to be related to the amount of learning in reward-based motor learning (Therrien et al. 2016, 2018).

Performance independent reward feedback, as was provided in our previous experiment (van Mastrigt et al. 2020), ensures independent samples of motor noise and exploration but does not allow the participant to improve performance. Hence this type of feedback is not suitable for studying the relation between exploration and changes in performance. In the ATTC method, the problem of selection bias on successful trials is solved by taking the trial preceding the (non-)successful trial as a reference trial to calculate trial-to-trial changes, as was also done by (Ranjan and Smith 2018). This is not commonly done in reward-based motor learning literature, so the variability estimates post-success reported by (Pekny et al. 2015; Therrien et al. 2018; Uehara et al. 2019; van der Kooij and Smeets 2018) may have been influenced by sample correlations between motor noise and exploration. In what way exactly, we do not know, however, because this influence seems to be related to the relative size of motor noise and exploration in the learners. Especially for the data of Uehara et al. (Uehara et al. 2019), where a decrease in exploration over a learning process was hypothesized (i.e., moving leftward in Fig. 6a when you assume motor noise to be constant), it would be nice to reanalyze the data to see whether sample correlations between motor noise and exploration on successful trials may have influenced the conclusion that exploration does not decrease over time.

The second pitfall that we identified is that trial-to-trial changes may or may not incorporate reference trial exploration, depending on reward history preceding the successful or non-successful trial. More exploration may be present in trials preceded by a non-successful trial than in trials preceded by a successful trial. Since some reference trials do and others do not contain exploration, variability is underestimated with a factor that depends on the amount of exploration draws contributing to a trial-to-trial change. By selecting trial sequences of specific trial outcomes and subtracting only estimates based on variability following double success from single failure, the amount of draws in the two resulting exploration estimates is constant and can thus be corrected for. The second pitfall implies that estimates of variability following success or failure that are based on all trial-to-trial changes rather than trial-to-trial changes following specific trial sequences, may be too low. This means that if one infers exploration from variability following successful and non-successful trials reported in the literature (e.g., Therrien et al. 2016; Uehara et al. 2019)), one might underestimate how much the participant explored.

We found the ATTC method to be a model-free way to estimate exploration, at least if exploration is based on a reward history of one trial. This is an advantage over estimating variability by fitting a model and finding the best fit variability parameters, as was done by (Cashaback et al. 2019; Therrien et al. 2016, 2018), since these parameters are tightly dependent on the model used and are thus more difficult to compare. Moreover, parameter values obtained from fitting a multi-parameter model to data may not be independent (Cheng and Sabes 2006; van der Vliet et al. 2018). Another advantage of the ATTC method is that the method is insensitive to the extent the learner uses the feedback to adjust her aim point with (i.e., the learning parameter of the model). The relation between the amount of learning over an experiment is less clear but does not seem to strongly affect exploration estimates Fig. 12 (Online Resource 6).

The difficulty in quantifying exploration is that we do not know exactly how humans learn from reward. There are indications that humans indeed take into account reward history to regulate exploratory variability, as described by the model of Dhawale et al. (2019): experimental data of (Cashaback et al. 2019; Holland et al. 2018; Pekny et al. 2015; Uehara et al. 2019) support the existence of a variability control function by showing higher trial-to-trial changes following trials with a lower reward frequency in the past three trials. Also, humans may take into account reward prediction errors rather than trial outcomes to gradually update the aim point with (Izawa and Shadmehr 2011; Palidis et al. 2019). Moreover, besides the four models that we used, other mechanisms of reward-based motor learning have also been proposed. For instance, learning may be a process based on the relative uncertainties of reward and sensory prediction errors (Izawa and Shadmehr 2011), a sequential decision-making process corrected for motor noise (Chen et al. 2017) or be some associative learning process linking probabilities of reward to motor actions as has recently has been hypothesized for error-based implicit learning (Avraham et al. 2020).

Two fascinating avenues for future research and possible threats to the validity of the ATTC method are the possibilities that exploration and motor noise are not independent, and that exploration is not randomly drawn as implemented in the four models that we used. It might make sense for exploration to vary systematically rather than randomly, possibly depending on the stage of learning (Abe and Sternad 2013; Dhawale et al. 2017; Sternad 2018). Systematic exploration is compatible with the finding that explicit processes contribute to reward-based motor learning", as suggested by Reviewer 2 upon approval of the paper (email of June 23th) (Codol et al. 2018; Holland et al. 2018).

In conclusion, we validated the additional trial-to-trial change (ATTC) method for models that regulate exploration based on previous binary trial outcome and on the way identified two pitfalls in quantifying exploration based on trial-to-trial changes. The first pitfall is that performance-dependent reward introduces negative covariation between samples of motor noise and exploration on successful trials. The second pitfall is that the presence or absence of reference trial exploration causes trial-to-trial changes to be underestimated. In the ATTC method, both pitfalls are circumvented by calculating trial-to-trial changes using triplets of trials rather than duos and by calculating and subtracting sets of trial-to-trial changes following specific reward sequences. This way, the ATTC method yields exploration estimates that are insensitive to learning, task and learner parameters for models that regulate exploration based on a reward history of only one trial.

## Supplementary Information

Below is the link to the electronic supplementary material.

Supplementary file1 (PDF 124 kb)

Supplementary file2 (PDF 922 kb)

Supplementary file3 (PDF 1598 kb)

Supplementary file4 (PDF 3422 kb)

## Data Availability

All data are available in the Open Science Foundation repository (https://osf.io/x7hp9/).

## Code availability

All scripts are available in the Open Science Foundation repository (https://osf.io/x7hp9/).
