# Supplementary material for: Pitfalls in quantifying exploration in reward-based motor learning and how to avoid them
Source: Biol Cybern. 2021 Aug 2;115(4):365–82. doi: 10.1007/s00422-021-00884-8 (PMC8382626; doi:10.1007/s00422-021-00884-8)
Supplement: Supplementary file 1 — Supplementary file1 (PDF 124 kb) [file 422_2021_884_MOESM1_ESM.pdf]

Supplementary information 1 - The Dhawale model

Exploratory variability control based on reward history

In the Dhawale19 model, exploration depends on the history of obtained rewards. In this model, reward history determines the variance of the distribution from which exploration is drawn ($\sigma_\eta^2(t)$). A history associated with (more) reward absence results in a higher exploratory variance than a history with (more) reward presence. Reward history of the τ previous trials determines the size of $\sigma_\eta^2(t)$. Reward history is calculated as the average reward rate on trial t ($\overline{R_\tau}^{(t)}$). In rats, Dhawale et al. (2019) estimated the time-scale τ to be 5 past trials. This so-called “inferred memory window for reinforcement on past trials” or “time-scale of the experimentally observed decay of the effect of single-trial outcomes on variability” (τ) influences the calculation of the average reward rate ($\overline{R_\tau}^{(t)}$) via a reward rate update fraction (β):

$$\beta \ = \ 1 - e^{\frac{-1}{\tau}}$$

Longer timescales τ are associated with smaller reward rate update fractions β. The reward rate update fraction β determines the weighting of the last obtained reward and the previous average reward rate estimate in the calculation of the newest average reward rate estimate. Smaller values of β result in more weight of the previous single-trial outcome $R^{(t-1)}$. Larger values of β result in more weight of the previous average reward rate estimate $\overline{R_\tau}^{(t-1)}$.

$$\overline{R_\tau}^{(t)} \ = \ \overline{R_\tau}^{(t-1)} + \beta * RPE^{(t-1)} = \ (1-\beta) * \ \overline{R_\tau}^{(t-1)} + \beta * R^{(t-1)}$$

If τ=0: β=1 $\qquad \overline{R_0}^{(t)} = (1-1) * \ \overline{R_0}^{(t-1)} + 1 * R^{(t-1)} = R^{(t-1)}$

If τ=∞:β=0 $\qquad \overline{R_\infty}^{(t)} = (1-0) * \ \overline{R_\infty}^{(t-1)} + 0 * R^{(t-1)} = \overline{R_\infty}^{(t-1)}$

If the time-scale of the decay of the effect of single-trial outcomes on variability is set to τ =0 there is no decay, i.e. β=1. This results in a model that estimates its previous average reward rate based on the previous reward only ($R^{(t-1)}$), which is the same as the Ther18 model.

References

Dhawale, A. K., Miyamoto, Y. R., Smith, M. A., & Ölveczky, B. P. (2019). Adaptive Regulation of Motor Variability. *Current Biology, 29*(21), 3551-3562.e7. https://doi.org/10.1016/j.cub.2019.08.052

Nina M. van Mastrigt, Katinka van der Kooij, Jeroen B.J. Smeets – Vrije Universiteit Amsterdam, Department of Human Movement Sciences – n.m.van.mastrigt@vu.nl
