# Supplementary material for: Pitfalls in quantifying exploration in reward-based motor learning and how to avoid them
Source: Biol Cybern. 2021 Aug 2;115(4):365–82. doi: 10.1007/s00422-021-00884-8 (PMC8382626; doi:10.1007/s00422-021-00884-8)
Supplement: Supplementary file 2 — Supplementary file2 (PDF 922 kb) [file 422_2021_884_MOESM2_ESM.pdf]

Supplementary information 4 Trial-to-trial changes in motor noise and exploration following success

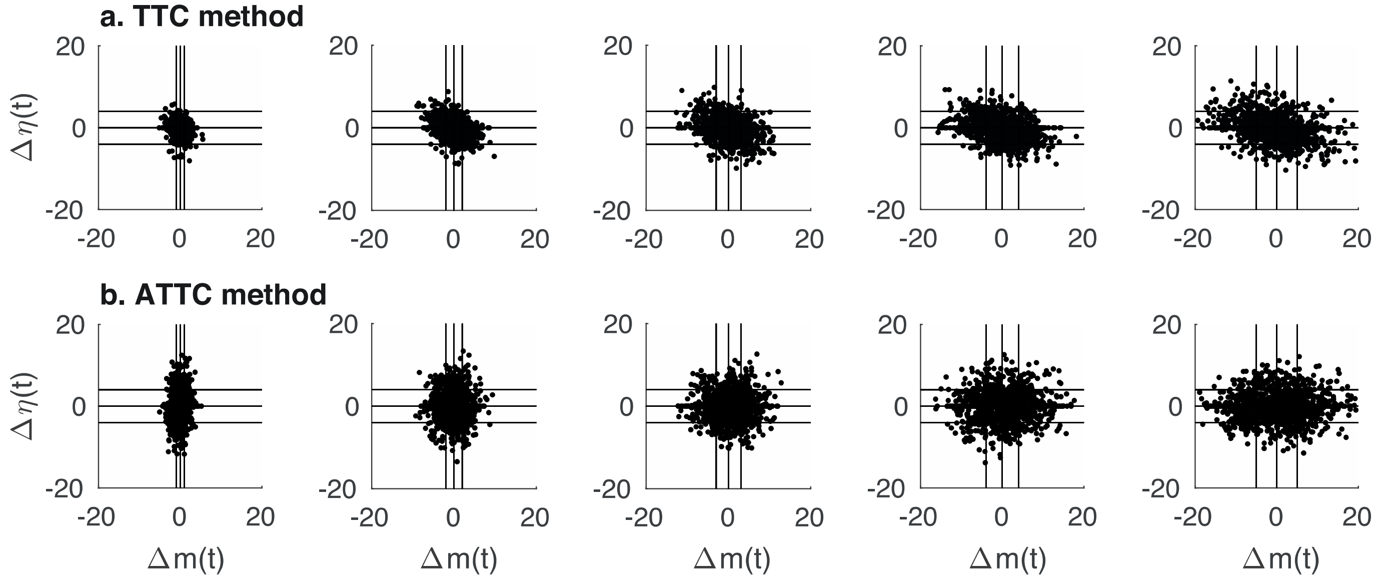

Fig. 11 Trial-to-trial changes in motor noise and exploration following success. Trial-to-trial changes in motor noise and exploration following success. Changes in draws of motor noise and exploration following successful trials. The first five simulations of a simulation set of the Therrien16 model, a random reward criterion and default exploration ($\sigma_{\eta*}^2 = 16$) have been plotted for increasing values of motor noise. Dotted lines indicate $\pm\sigma$
