# Supplementary material for: Pitfalls in quantifying exploration in reward-based motor learning and how to avoid them
Source: Biol Cybern. 2021 Aug 2;115(4):365–82. doi: 10.1007/s00422-021-00884-8 (PMC8382626; doi:10.1007/s00422-021-00884-8)
Supplement: Supplementary file 3 — Supplementary file3 (PDF 1598 kb) [file 422_2021_884_MOESM3_ESM.pdf]

Supplementary information 6 Relation between learning and similarity ratio

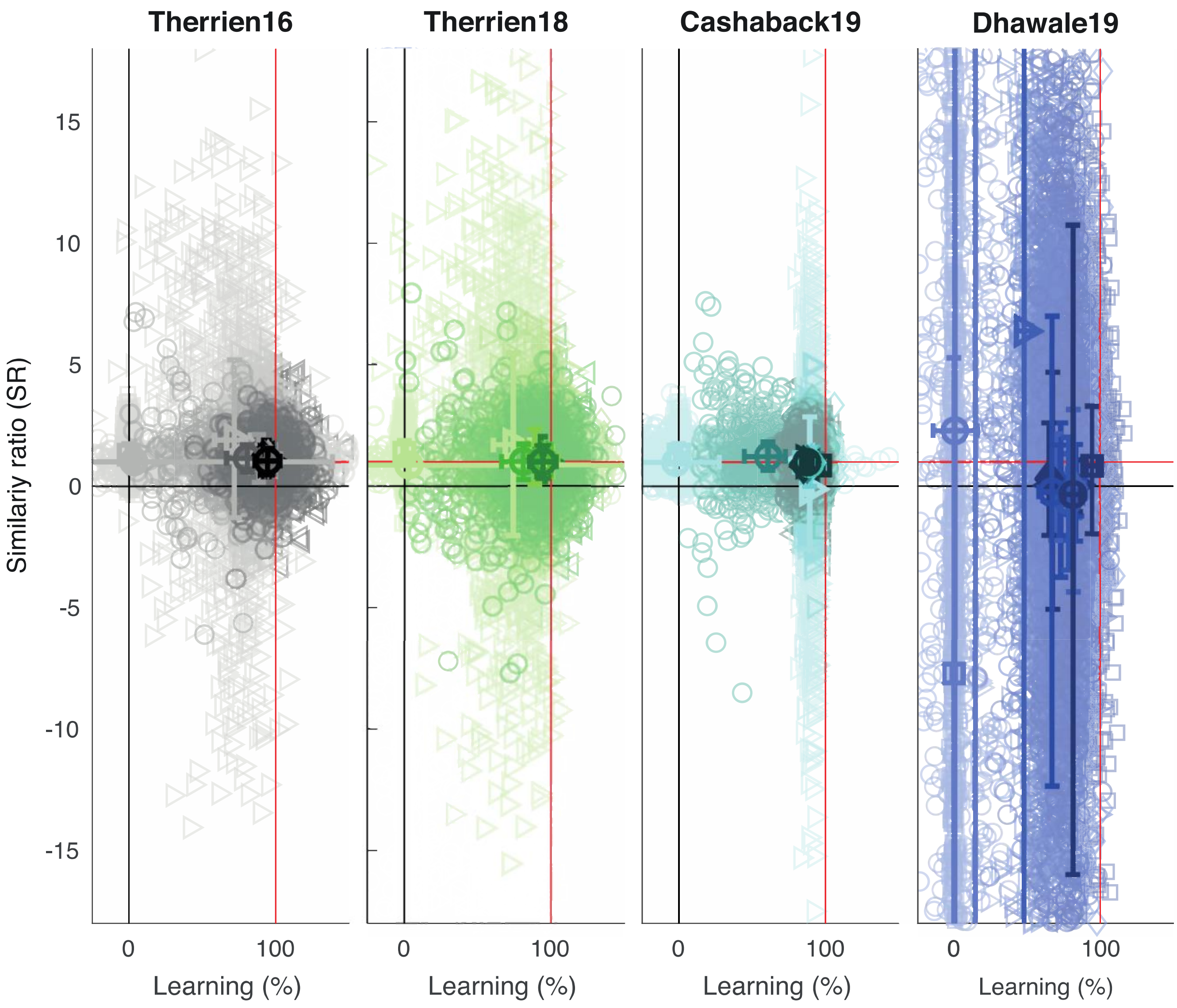

Fig. 12 Relation between learning and similarity ratio. The adaptive reward criterion is used and estimates are calculated with the ATTC method. For each model, the similarity ratio between estimated exploration and the exploration that was actually present in the model is plotted against the learning achieved in each simulation. Symbols with error bars depict the median and interquartile range of simulations of a simulation set. Symbols and colors are in accordance with Fig. 5 and Fig. 9. Horizontal red line indicates perfect exploration estimation. Vertical red line indicates full learning
