# Supplementary material for: Pitfalls in quantifying exploration in reward-based motor learning and how to avoid them
Source: Biol Cybern. 2021 Aug 2;115(4):365–82. doi: 10.1007/s00422-021-00884-8 (PMC8382626; doi:10.1007/s00422-021-00884-8)
Supplement: Supplementary file 4 — Supplementary file4 (PDF 3422 kb) [file 422_2021_884_MOESM4_ESM.pdf]

Supplementary information 1 - The Dhawale model

Exploratory variability control based on reward history

In the Dhawale19 model, exploration depends on the history of obtained rewards. In this model, reward history determines the variance of the distribution from which exploration is drawn ($\sigma_\eta^2(t)$). A history associated with (more) reward absence results in a higher exploratory variance than a history with (more) reward presence. Reward history of the τ previous trials determines the size of $\sigma_\eta^2(t)$. Reward history is calculated as the average reward rate on trial t ($\overline{R_\tau}^{(t)}$). In rats, Dhawale et al. (2019) estimated the time-scale τ to be 5 past trials. This so-called "inferred memory window for reinforcement on past trials" or "time-scale of the experimentally observed decay of the effect of single-trial outcomes on variability" (τ) influences the calculation of the average reward rate ($\overline{R_\tau}^{(t)}$) via a reward rate update fraction (β):

$$\beta = 1 - e^{\frac{-1}{\tau}}$$

Longer timescales τ are associated with smaller reward rate update fractions β. The reward rate update fraction β determines the weighting of the last obtained reward and the previous average reward rate estimate in the calculation of the newest average reward rate estimate. Smaller values of β result in more weight of the previous single-trial outcome $R^{(t-1)}$. Larger values of β result in more weight of the previous average reward rate estimate $\overline{R_\tau}^{(t-1)}$.

$$\overline{R_\tau}^{(t)} = \overline{R_\tau}^{(t-1)} + \beta * RPE^{(t-1)} = (1-\beta) * \overline{R_\tau}^{(t-1)} + \beta * R^{(t-1)}$$

If τ=0: β=1 $\quad \overline{R_0}^{(t)} = (1-1) * \overline{R_0}^{(t-1)} + 1 * R^{(t-1)} = R^{(t-1)}$

If τ=∞: β=0 $\quad \overline{R_\infty}^{(t)} = (1-0) * \overline{R_\infty}^{(t-1)} + 0 * R^{(t-1)} = \overline{R_\infty}^{(t-1)}$

If the time-scale of the decay of the effect of single-trial outcomes on variability is set to τ =0 there is no decay, i.e. β=1. This results in a model that estimates its previous average reward rate based on the previous reward only ($R^{(t-1)}$), which is the same as the Ther18 model.

References

Dhawale, A. K., Miyamoto, Y. R., Smith, M. A., & Ölveczky, B. P. (2019). Adaptive Regulation of Motor Variability. *Current Biology, 29*(21), 3551-3562.e7. https://doi.org/10.1016/j.cub.2019.08.052

Nina M. van Mastrigt, Katinka van der Kooij, Jeroen B.J. Smeets – Vrije Universiteit Amsterdam, Department of Human Movement Sciences – n.m.van.mastrigt@vu.nl

Supplementary information 2 Model parameters derived from the literature

Table 4 Model parameters derived from the literature.

Parameter	Therrien et al. (2016)	Therrien et al. (2018)		Cashaback et al. (2019)			Dhawale et al. (2019)
**Derived from:**	Fig 7, Healthy young	**Experiment 1,** Fig 7, Healthy young	**Experiment 2,** Fig 7, Healthy young	**Experimental estimate**, S2 Data	**Simulation best fit**, S2 Data	**Simulation parameter variations**, Fig 8B	**Experimental estimate**, Fig 2D, Fig 3D
$\boldsymbol{\sigma_m^2}$	6.8°<br>(0.04 – 25)	4°<br>(0 – 16)	4°<br>(0 – 16)	0.98*$\sigma^2_{total,baseline}$<br>(0.86-1.10)	0.44*$\sigma^2_{total,baseline}$<br>(0.40-0.94)	1.49 – 2.30°	14°
$\boldsymbol{\sigma_\eta^2}$	4.0°<br>(0.3 – 12.3)	$R^{(t-1)}$ | σ_η^2<br>1 | 3.6 (0 – 49)<br>0 | 22.1 (0 – 81)	$R^{(t-1)}$ | σ_η^2<br>1 | 1.7 (0 – 9)<br>0 | 28.1 (1 – 324)	0.72*$\sigma^2_{total,baseline}$<br>(0.69-0.75)	0.66*$\sigma^2_{total,baseline}$<br>(0.66-1.04)	1.49 – 2.30°	$\overline{R_5}^{(t)}$ | σ_η^2<br>0 | 19.5°<br>0.16 | 9.7°<br>0.33 | 5.5°<br>0.50 | 3.5°<br>0.66 | 2.1°<br>0.83 | 1°<br>1 | 0°
$\boldsymbol{\alpha}$	-	-		0.40*$\sigma_{total,baseline}$ (0.25-0.63)			0.23 +/- 0.19
**Target amplitude** (fraction of σ_m)	± 6*σ_m	± 8*σ_m		± 3-6*$\sigma_{total,baseline}$			± 0.5-5*σ_m
**Reward criterion (R = 1 if:)**	Fixed:<br>target ± 5.75°			Fixed:<br>-3 – 6*$\sigma_{total,baseline}$			Fixed:<br>target ± 2.3°
	Adaptive:<br>$\overline{EP}_{t-1:t-10}$ ≤ EP ≤ target + 5.75°	Adaptive:<br>$\overline{EP}_{t-1:t-10}$ ≤ EP ≤ target + 5.75°					

References

Cashaback, J. G. A., Lao, C. K., Palidis, D. J., Coltman, S. K., McGregor, H. R., & Gribble, P. L. (2019). The gradient of the reinforcement landscape influences sensorimotor learning. *PLoS Computational Biology*, *15*(3), e1006839. https://doi.org/10.1371/journal.pcbi.1006839

Dhawale, A. K., Miyamoto, Y. R., Smith, M. A., & Ölveczky, B. P. (2019). Adaptive Regulation of Motor Variability. *Current Biology*, *29*(21), 3551-3562.e7. https://doi.org/10.1016/j.cub.2019.08.052

Therrien, A. S., Wolpert, D. M., & Bastian, A. J. (2016). Effective Reinforcement learning following cerebellar damage requires a balance between exploration and motor noise. *Brain*, *139*(1), 101–114. https://doi.org/10.1093/brain/awv329

Therrien, A. S., Wolpert, D. M., & Bastian, A. J. (2018). Increasing Motor Noise Impairs Reinforcement Learning in Healthy Individuals. *Eneuro*, *5*(3), e0050-18.2018. https://doi.org/10.1523/ENEURO.0050-18.2018

Supplementary information 3 Learning

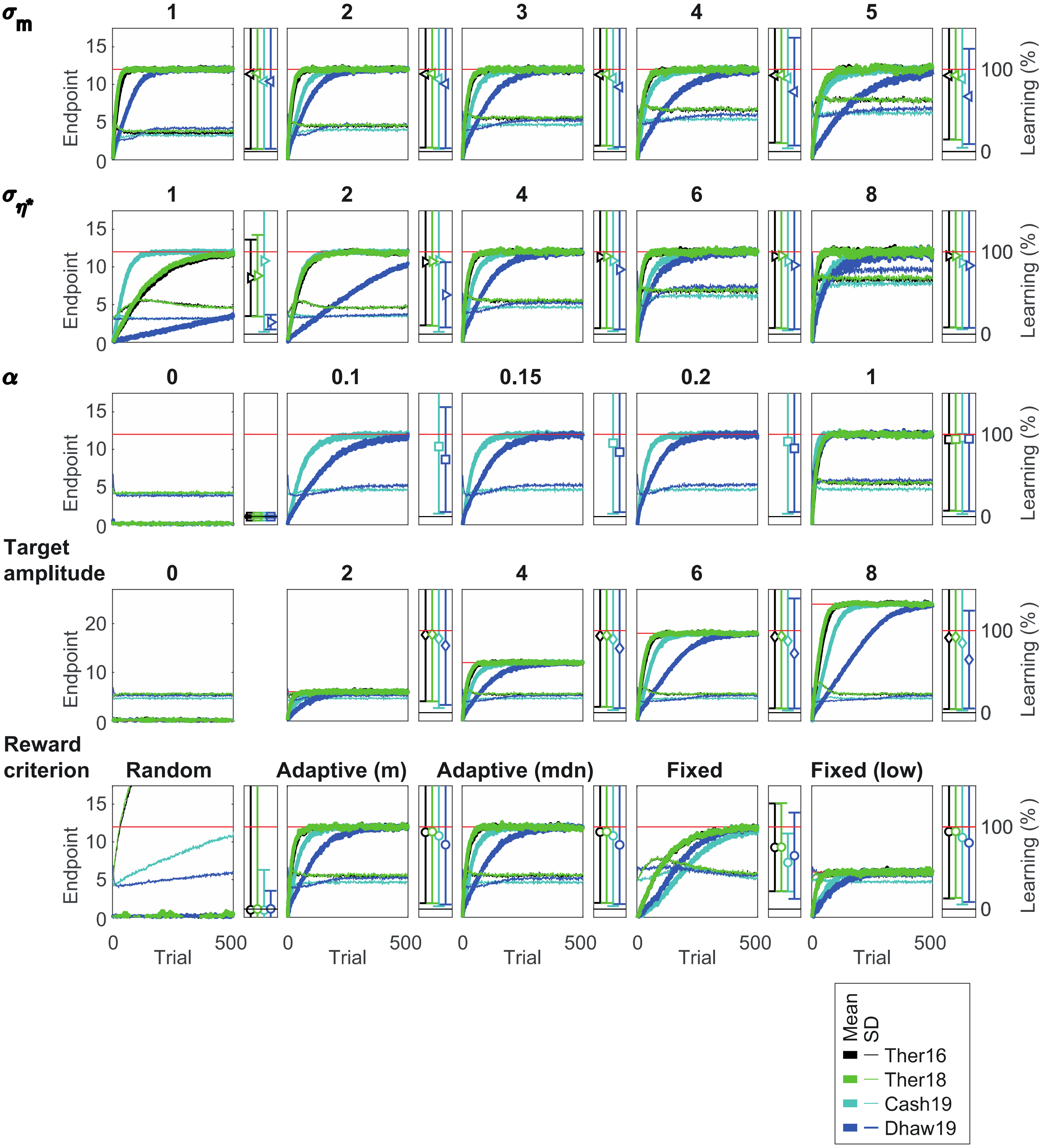

Fig. 10 Learning. Each row displays learning for variations of one parameter, with the other parameters set to default. Learning curves (wide panels) and learning (narrow panels) are averaged over the 1000 simulations within each simulation set for each model. Wide panels: Average (thick lines) and standard deviation (thin lines) of the endpoint over time. Red horizontal lines indicate target amplitude. Note that vertical axes are equal in the top three rows but not the lower two rows. Also note that because the Ther16

and Ther18 models do not contain a learning parameter, it is effectively set to $\alpha = 1$. This is why curves for these models are lacking in the third row, and why these models show faster learning in the other rows. Narrow panels: Average learning over simulations. Error bars indicate standard deviations. Red horizontal lines indicate full learning. Also note that the calculation of learning is impossible when the target amplitude is zero, which is why a panel is missing on the fourth row

Supplementary information 4 Trial-to-trial changes in motor noise and exploration following success

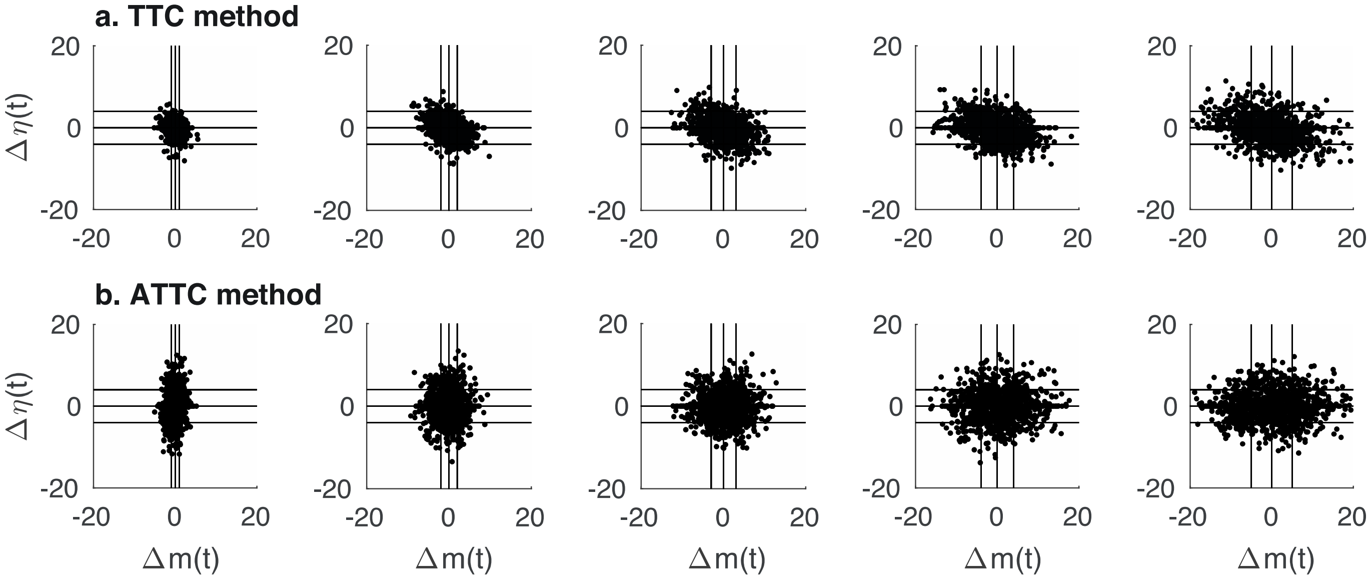

Fig. 11 Trial-to-trial changes in motor noise and exploration following success. Trial-to-trial changes in motor noise and exploration following success. Changes in draws of motor noise and exploration following successful trials. The first five simulations of a simulation set of the Therrien16 model, a random reward criterion and default exploration ($\sigma_{\eta*}^2 = 16$) have been plotted for increasing values of motor noise. Dotted lines indicate $\pm\sigma$

Supplementary information 5 Variability estimation

In the (A)TTC method, we use trial-to-trial changes to estimate variability with. These trial-to-trial change-based variability estimates have to be converted to variances.

Variance of a variable and variance of changes in that variable

The variance of a variable is calculated from a set of observations of that variable. The variance of trial-to-trial changes in that variable is twice as large as the variance of the variable itself.

Correction of trial-to-trial based variability estimates

In the TTC method and ATTC method we use different correction factors to convert variability estimates based on trial-to-trial changes to variances. Both methods estimate variability based on the median of squared trial-to-trial changes.

TTC method

In the TTC method, the median of squared trial-to-trial changes ($\widetilde{\Delta^2}$) is converted to variances (σ^2) using the correction factor $b_{TH} = {}^{\pi}/_{4} \approx 0.79$. This value is based on the relation between the mean amplitude of trial-to-trial changes and standard deviation as reported by Thirey & Hickman (2015): $\overline{|\Delta|} = \frac{2}{\sqrt{\pi}}\sigma$. This relation holds for a one-dimensional process in which each trial is defined by a random draw from N(0, σ^2). Each trial-to-trial change is hence the difference between two draws.

$$\sigma^2 = \frac{\pi}{4} \cdot \overline{|\Delta|}^2 \approx \frac{\pi}{4} \cdot \widetilde{\Delta^2}$$

ATTC method

In the ATTC method, the median of squared trial-to-trial changes ($\widetilde{\Delta^2}$) is converted to variances (σ^2) using the correction factor $b_{TH} \approx 2.2$. This value is obtained by simulating sequences of random draws from N(0, σ^2) and calculating the median of squared trial-to-trial changes from it. Each trial-to-trial change is calculated as the trial draw minus zero. Each trial-to-trial change thus corresponds to one draw.

$$\sigma^2 = 2.2 \cdot \widetilde{\Delta^2}$$

Nina M. van Mastrigt, Katinka van der Kooij, Jeroen B.J. Smeets – Vrije Universiteit Amsterdam, Department of Human Movement Sciences – n.m.van.mastrigt@vu.nl

Matlab script

```
% Explanation and calculation of correction factors
Ntrials = 5000;
Nsim = 10000;
sd_in = 2;
var_in = sd_in^2;
rng('Shuffle')

%% Generate random draws of exploration
for s = 1:Nsim
    randseq_eta(1:Ntrials,s) = normrnd(0,sd_in,Ntrials, 1); %NtrialsxNsim
end

%% Calculate trial-to-trial changes (=delta EP=dep): all trials contain
exploration.
%Trial-to-trial change consists of 2 draws.
dep_00=randseq_eta(2:Ntrials,:)-randseq_eta(1:Ntrials-1,:);

% Is variance of CHANGES in eta indeed twice as large as variance of eta
% (Cashaback 2019, S2)?
% ANSWER: YES.
sd_dep00 = mean(std(dep_00,0,1)); %SD over trials in sim. Mean over sims.
var_dep00 = sd_dep00^2;
perf_var_dep00_shouldbe2 = var_dep00/var_in % var_dep00=2*var00 is true.

% Is relation between mean absolute delta_EP and SD indeed as in
% Thirey-Hickman (2015)? Their delta_EP also contain 2 draws.
% ANSWER: YES.
meanabs_dep00 = mean(mean(abs(dep_00),1)); %Mean abs delta, mean over sims.
perf_meanabs_dep00_shouldbe1p12 = meanabs_dep00/sd
%meanabs_dep00/sd=2/sqrt(pi)=1.1284 is true.
perf_TH_shouldbe0p78 = 1/perf_meanabs_dep00_shouldbe1p12^2 % Squared.

% What is the relation between our estimate of variability for this type of
% trial-to-trial changes (that contain 2 draws) and variance of eta?
% ANSWER: median of squared trial-to-trial changes = 0.91 * var_in
sqmed_dep00 = mean(median(dep_00.^2,1)); %Mdn over trials, mean over sims.
perf_sqmed_dep00_dontknowyet = sqmed_dep00/var_in %Our delta measure yields about
0.91 of the original variance.

%% Calculate trial-to-trial changes (delta EP) between trials with exploration
and zero.
% Trial-to-trial change consists of 1 draw, other one is zero.
dep_10=randseq_eta(2:Ntrials,:)-zeros(Ntrials-1,Nsim);

% Is variance of CHANGES between eta and zero indeed the variance of eta?
% ANSWER: YES.
sd_dep10 = mean(std(dep_10,0,1)); %SD over trials in sim. Mean over sims.
var_dep10 = sd_dep10^2;
perf_var_dep10_shouldbe1 =var_dep10/var_in

% What is the relation between our estimate of variability for this type of
% trial-to-trial changes (that contain 1 draw) and variance of eta?
% ANSWER: median of squared trial-to-trial changes = 0.45 * var_in
sqmed_dep10 = mean(median(dep_10.^2,1));
perf_sqmed_dep10_dontknowyet =sqmed_dep10/var_in %Our delta measure yields about
0.45 of the original variance.
```


Nina M. van Mastrigt, Katinka van der Kooij, Jeroen B.J. Smeets – Vrije Universiteit Amsterdam, Department of Human Movement Sciences – n.m.van.mastrigt@vu.nl

```
%% Correction factors for 2 draws vs 1 draw: d_EP estimate to variance.
b_2draws = 1/perf_sqmed_dep00_dontknowyet %1.09
b_1draw = 1/perf_sqmed_dep10_dontknowyet %2.18
b_TH_2draws = 1/(perf_meanabs_dep00_shouldbe1p12^2) %Thirey Hickman, 0.79

%% Evaluation
% Use b_1draw in ATTC method.
% The Thirey-Hickman factor (0.78) and the b_corr_2draws (1.09) differ
% while they are based on the same trial-to-trial changes with 2 draws.
% So part of the underestimation of the TTC method was caused by the
% fact that mean(abs(deltas))^2 does not approximate median(deltas^2) well.
```

References

Thirey, B., & Hickman, R. (2015). *Distribution of Euclidean Distances Between Randomly Distributed Gaussian Points in n-Space*. http://arxiv.org/abs/1508.02238

Supplementary information 6 Relation between learning and similarity ratio

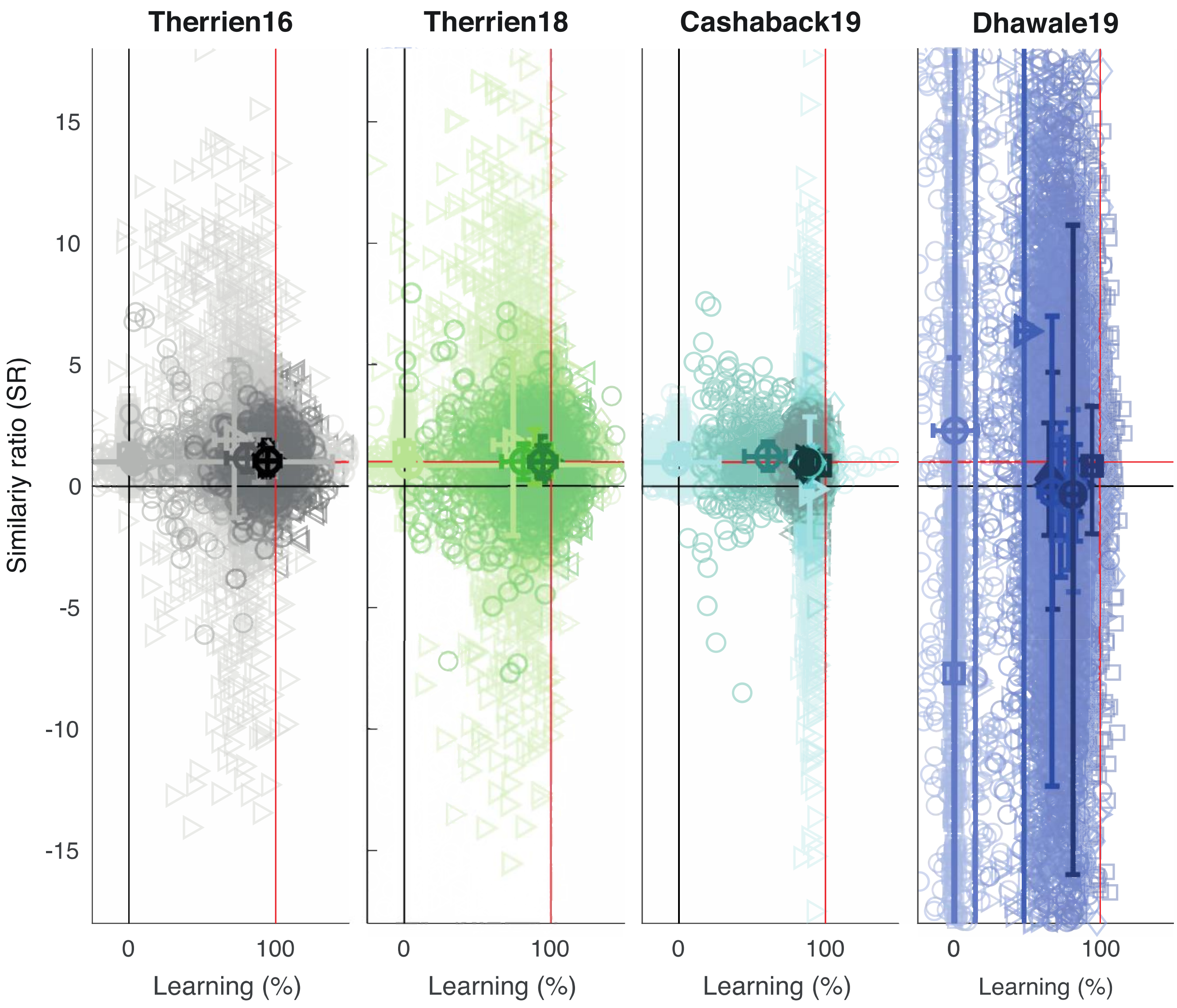

Fig. 12 Relation between learning and similarity ratio. The adaptive reward criterion is used and estimates are calculated with the ATTC method. For each model, the similarity ratio between estimated exploration and the exploration that was actually present in the model is plotted against the learning achieved in each simulation. Symbols with error bars depict the median and interquartile range of simulations of a simulation set. Symbols and colors are in accordance with Fig. 5 and Fig. 9. Horizontal red line indicates perfect exploration estimation. Vertical red line indicates full learning
